# Development of lisinopril radioconjugates for nuclear imaging of the angiotensin converting enzyme

**DOI:** 10.1007/s00259-025-07386-w

**Published:** 2025-06-07

**Authors:** Christian Vaccarin, Darja Beyer, Anzhelika Moiseeva, Chiara Favaretto, Nicholas P. van der Meulen, Benjamin D. Hunkeler, Niels J. Rupp, Giovanni Marzaro, Roger Schibli, Cristina Müller

**Affiliations:** 1 Center for Radiopharmaceutical Sciences, PSI Center for Life Sciences, Villigen-PSI, Switzerland; 2 Nuclear Medicine Department, University Hospital Basel, Basel, Switzerland; 3 Laboratory of Radiochemistry, PSI Center for Energy and Nuclear Safety, Villigen-PSI, Switzerland; 4 Department of Pathology and Molecular Pathology, University Hospital Zurich, Zurich, Switzerland; 5 Faculty of Medicine, University of Zurich, Zurich, Switzerland; 6 Department of Diagnostics and Public Health, University of Verona, Verona, Italy; 7 Department of Chemistry and Applied Biosciences, ETH Zurich, Zurich, Switzerland

**Keywords:** Angiotensin converting enzyme, Lisinopril, Gallium-67, Gallium-68, Scandium-44, PET imaging

## Abstract

**Purpose:**

The angiotensin converting enzyme (ACE) is essential in maintaining cardiovascular homeostasis and its dysfunction is associated with various pathological conditions. This study aimed to investigate lisinopril-based radioconjugates for nuclear imaging of ACE.

**Methods:**

Lisinopril conjugates were prepared using solid-phase peptide synthesis and labeled with gallium-67. The resulting radioconjugates were assessed for their in vitro stability in saline and in mouse and human blood plasma. ACE-mediated uptake was evaluated in HEK cells transfected with human ACE, and the findings were interpreted using molecular dynamic studies. Tissue distribution profiles of the radioconjugates were evaluated in mice bearing HEK-ACE xenografts by means of nuclear imaging and classic biodistribution studies. The feasibility to detect physiologic ACE was further assessed by ex vivo autoradiography of the lungs and the kidneys collected from mice injected with [^67^Ga]Ga-DOTA-LIS-02.

**Results:**

DOTA-LIS-01, DOTA-LIS-02 and NODAGA-LIS-02 were prepared with > 98% chemical purity. Radiolabeling of the conjugates with gallium-67 was achieved at 80 MBq/nmol with > 99% radiochemical purity. [^67^Ga]Ga-DOTA-LIS-02 and [^67^Ga]Ga-NODAGA-LIS-02 exhibited radiolytic stability for up to 3 h in saline and remained intact in mouse and human blood plasma for 1 h. The uptake of [^67^Ga]Ga-DOTA-LIS-02 and [^67^Ga]Ga-NODAGA-LIS-02 in HEK-ACE cells after 3 h incubation reached ~ 57% and ~ 50%, respectively, while only ~ 21% cell uptake was reached with [^67^Ga]Ga-DOTA-LIS-01. This discrepancy was ascribed to the favorable binding mode of the LIS-02 radioconjugates with a longer linker between DOTA and lisinopril, as demonstrated by molecular dynamic studies. Nuclear images demonstrated uptake of all radioconjugates in HEK-ACE xenografts but not in HEK-ACE2 xenografts. At 1 h after injection of [^67^Ga]Ga-DOTA-LIS-02, the accumulation in HEK-ACE xenografts of mice reached 14 ± 3% IA/g, whereas only 3.6 ± 0.3% IA/g uptake was observed for [^67^Ga]Ga-NODAGA-LIS-02. The ex vivo autoradiograms of kidneys and lungs of mice injected with [^67^Ga]Ga-DOTA-LIS-02 only, or co-injected with excess lisinopril confirmed ACE-specific binding of this radioconjugate.

**Conclusion:**

[^67^Ga]Ga-DOTA-LIS-02 emerged as the most promising candidate to visualize ACE in mice. Further (pre)clinical studies will be necessary to validate the radioconjugate’s potential for assessing ACE expression dynamics under pathophysiological conditions.

**Graphical abstract:**

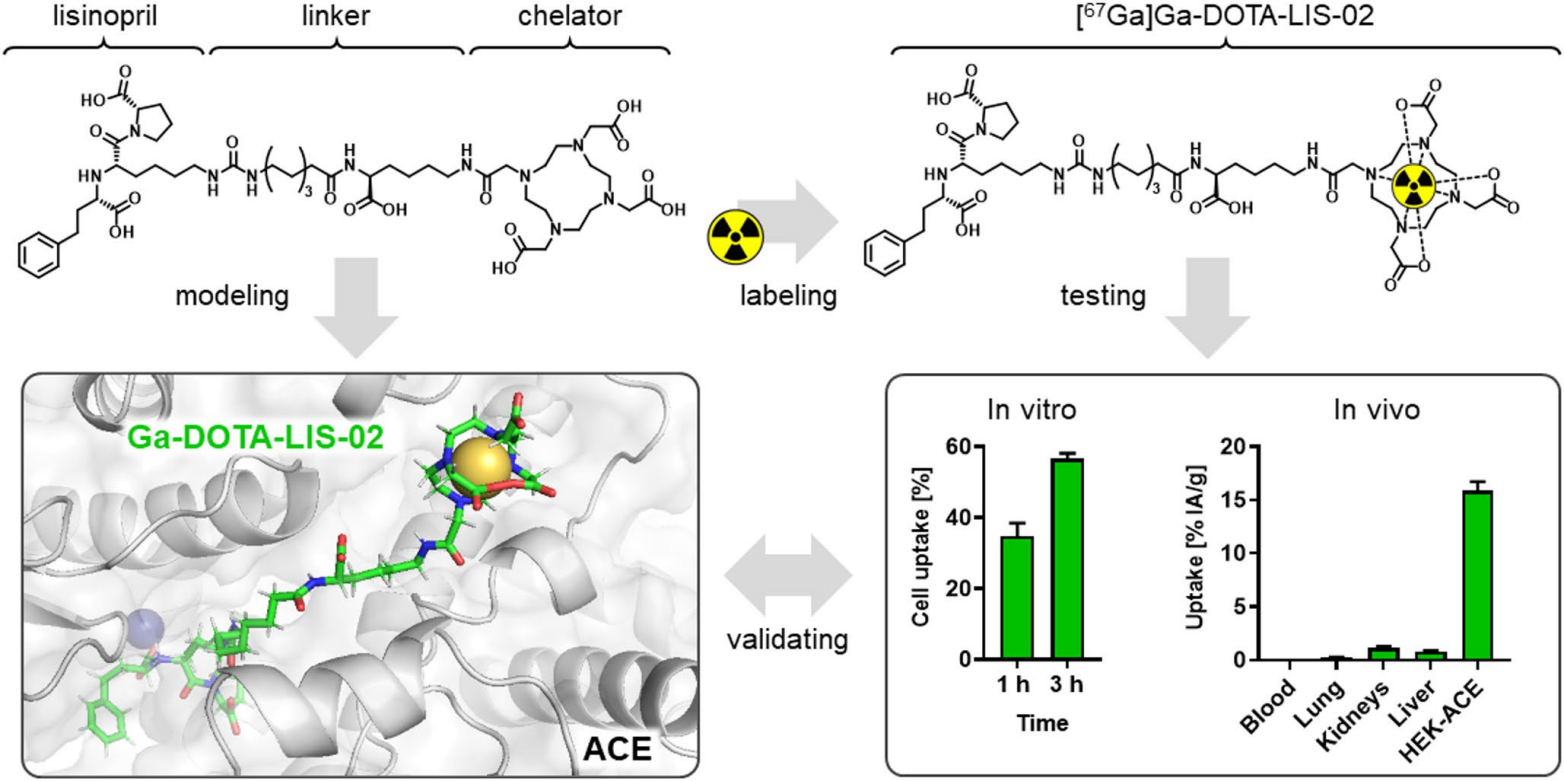

**Supplementary Information:**

The online version contains supplementary material available at 10.1007/s00259-025-07386-w.

## Introduction

The renin-angiotensin-aldosterone system (RAAS) is essential for maintaining cardiovascular homeostasis. Within this system, the carboxypeptidase angiotensin converting enzyme (ACE) facilitates the conversion of angiotensin I to angiotensin II which acts as a vasoconstrictor as part of normal physiological processes [1]. ACE is expressed in various epithelial tissues, including the vasculature, heart, lungs, intestines and kidneys, where it mediates both a vasoconstrictive and proinflammatory effect [2]. In many pathologies, including high blood pressure, heart failure and neurodegenerative diseases, dysregulation of the activity and/or expression of ACE has been reported [3, 4]. The role of ACE and ACE inhibitors in the context of Covid-19 and related symptoms has been extensively discussed in the literature [5–7]. As part of the RAAS, the angiotensin converting enzyme-2 (ACE2), known as the entry receptor for SARS-CoV-2 [8], has counterregulatory function to ACE [9, 10]. Investigating the interplay between ACE and ACE2 would, thus, offer valuable insights into the mechanisms underlying the known symptoms of cardiovascular dysregulation in Covid-19 as well as other cardiovascular diseases.

Efforts have been directed towards developing non-invasive imaging tools for the detection and quantification of ACE and ACE2 in order to explore their functions under (patho)physiological conditions [11, 12]. Previously, our group reported on the synthesis and preclinical evaluation of the radiopeptides [^67^Ga]Ga-DOTA-BPP9a and [^67^Ga]Ga-HBED-DX600, for nuclear imaging of ACE and ACE2, respectively [13]. [^67^Ga]Ga-DOTA-BPP9a, based on the ACE inhibitor teprotide [14, 15], was characterized by only moderate uptake in ACE-expressing tissue while off-target accumulation was high. Others developed small ligands based on known ACE inhibitors for labeling with fluorine-18 or technetium-99m, which enabled their use in preclinical imaging studies using positron emission tomography (PET) and single photon emission computed tomography (SPECT), respectively [16]. The 4-*cis*-[^18^F]fluorocaptopril ([^18^F]F-CAP) was the first clinically employed radiotracer for ACE imaging [17]. It served for the investigation of ACE expression in patients with pulmonary hypertension [16]. Indeed, [¹⁸F]F-CAP enabled defining the optimal dose of ACE inhibitor which was necessary to effectively block pulmonary ACE activity and, consequently, prevent the onset of pulmonary vascular remodeling [18]. These clinical studies underscore the translational potential of ACE-targeting radiotracers. The reactive thiol group of [¹⁸F]F-CAP was, however, shown to form dimers and mixed disulfides with endogenous proteins containing sulfhydryl groups which emerged as a significant limitation of this radiotracer [19]. Other ACE inhibitors, including lisinopril, have a higher binding affinity for tissue ACE, making them better candidates for the development of nuclear imaging agents [20]. One of the first lisinopril-based radioconjugates was designed by acylation of its primary amine with [^18^F]fluorobenzoic acid as a prosthetic group [21]. The resulting radiotracer proved to be a valuable tool for the in vitro quantification of ACE levels in tissue samples from ischemic cardiomyopathy and patients with heart failure [20] [^99m^Tc]Tc-tricarbonyl-based lisinopril radiotracers investigated for preclinical imaging of ACE in normal and transgenic rats for human ACE showed, however, substantial hepatobiliary excretion with high background accumulation in the liver and intestinal tract [16, 22].

The aim of this study was to develop lisinopril-based conjugates with a DOTA or NODAGA chelator for radiometal complexation. While such macrocyclic chelators enable the coordination of a wide range of radionuclides, we herein used gallium-67 (T_1/2_ = 3.26 d; Eγ = 93 keV, 185 keV, 300 keV) for preclinical SPECT imaging as a surrogate for the clinically used PET nuclide gallium-68 (T_1/2_ = 68 min; Eβ^+^_mean_ = 830 keV). In addition, we tested the utility of the longer-lived scandium-44 (T_1/2_ = 4.04 h; Eβ^+^_mean_ = 630 keV), potentially enabling PET imaging at later timepoints. The resultant radioconjugates were extensively evaluated in vitro and in xenograft-bearing mice.

## Methods

### Synthesis of DOTA-LIS-01, DOTA-LIS-02 and NODAGA-LIS-02

**Scheme 1:**
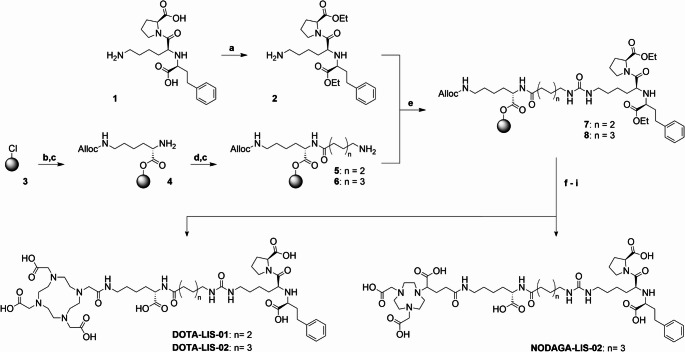
Synthesis scheme of the preparation of DOTA-LIS-01, DOTA-LIS-02 and NODAGA-LIS-02. Reaction conditions: **a**) Ethanol, SOCl_2_, reflux, overnight; **b**) Fmoc-Lys(Alloc)-OH, diisopropylethylamine (DIPEA), dichloromethane (DCM), room temperature (RT), overnight; **c**) 50% (*v/v*) piperidine in dimethylformamide (DMF), RT, 2 × 5 min; **d**) Fmoc-Ahx-OH or Fmoc-Aoc-OH, hexafluorophosphate benzotriazoletetramethyl uronium (HBTU), DIPEA, DMF, RT, 1 h; **e**) Triphosgene, DIPEA, DCM, 0 °C to RT, overnight; **f**) Pd(PPh_3_)_4_, morpholine, DCM, RT, 1 h; **g**) DOTA-tri(^t^Bu) ester or NODAGA-tri(^t^Bu) ester, HBTU, DIPEA, DMF, RT, 1 h; **h**) Trifluoroacetic acid, triisopropylsilane, Milli-Q water, RT, 2 h; **i**) LiOH, Milli-Q water, acetonitrile, RT, 7 h

DOTA-LIS-01, DOTA-LIS-02 and NODAGA-LIS-02 were synthesized using solid-phase peptide synthesis methodologies (Scheme 1). As a first step, both carboxylic functions of lisinopril (**1**) were esterified with ethyl groups for protection to obtain compound **2**. In parallel, the 2-chlorotrityl chloride resin (**3**) was loaded with Fmoc-Lys(Alloc)-OH. Following the cleavage of the Fmoc-protecting group, the resulting intermediate **4** was functionalized with an aminohexanoic acid (**5**) or aminooctanoic acid (**6**) linker. Subsequently, the lisinopril diethyl ester (**2**) was transformed in situ into the respective isocyanate, which was immediately reacted with the resin-immobilized intermediates to form the desired urea linkage (**7** and **8**). After cleavage of the Alloc-protecting group, the primary amino function was conjugated to a DOTA-tris(^t^Bu) ester to obtain DOTA-LIS-01 and DOTA-LIS-02 or to a NODAGA-tris(^t^Bu) ester to obtain NODAGA-LIS-02. Cleavage from the resin and global deprotection yielded the desired crude lisinopril conjugates. After purification using semipreparative high-performance liquid chromatography (HPLC), the chemical purity and identity of the compounds were determined by analytical HPLC and high-resolution mass spectrometry (HRMS), respectively (Supplementary Material).

### Preparation and stability of ^67^Ga- and ^44^Sc-labeled lisinopril conjugates

Gallium-67 (no-carrier-added [^67^Ga]GaCl_3_ in ~ 0.1 M HCl) was purchased from Curium Netherlands B.V. (the Netherlands, via b.e.imaging GmbH, Switzerland) and reprocessed according to a previously published method to enable radiolabeling at the indicated molar activities [23]. [^67^Ga]GaCl_3_ in HCl (0.1 M) was added to a sodium acetate solution (0.5 M) to obtain a buffered system of pH ~ 4. DOTA-LIS-01, DOTA-LIS-02 or NODAGA-LIS-02 was added from an aqueous stock solution (1 mM) to obtain a defined molar activity (5‒80 MBq/nmol) followed by incubation of the reaction mixture at 95 °C for 10 min. Scandium-44 (no-carrier-added [^44^Sc]Sc(OAc)_3_ in 0.5 M ammonium acetate, adjusted to pH 4 with acetic acid) was produced at Paul Scherrer Institute by the “Radionuclide Development” group. Radiolabeling was performed by direct addition of DOTA-LIS-02 from an aqueous stock solution (1 mM) to the ^44^Sc solution to obtain a defined molar activity (3‒20 MBq/nmol) followed by incubation at 95 °C for 10 min. Quality control was performed using a Merck Hitachi LaChrom HPLC system connected to a C18 column (Xterra^™^, 5 μm, 4.6 × 150 mm, Waters, Milford, MA, USA). The radioconjugates were eluted using a linear gradient of acetonitrile (5–80%) and Milli-Q water containing 0.1% (*v/v*) trifluoroacetic acid (95–20%) over 15 min at a flow rate of 1.0 mL/min (Supplementary Material).

The radiolytic stability of the radioconjugates (20 MBq/nmol) was investigated at an activity concentration of 10 MBq/100 µL in saline over 24 h at room temperature (Supplementary Material). HPLC analyses were performed immediately after radiolabeling (t = 0) and at t = 1 h, t = 3 h and t = 24 h after preparation of the radioconjugates using analytical HPLC as described above. Additionally, the stability of [^67^Ga]Ga-DOTA-LIS-02 and [^67^Ga]Ga-NODAGA-LIS-02 (6 MBq/nmol) was tested in mouse and human blood plasma, as well as in mouse liver and kidney homogenates for up to 3 h, to mimic the in vivo situation. The radioconjugates’ integrity was analysed using thin-layer chromatography and compared with the radioconjugates incubated in saline (Supplementary Material).

### *n*-Octanol/PBS distribution coefficients and serum protein-binding properties

The *n*-octanol/phosphate buffered saline (PBS) distribution coefficients (logD values) of the radioconjugates (20 MBq/nmol) were determined using the shake-flask method, as previously reported (Supplementary material) [13]. The binding affinity of the radioconjugates (20 MBq/nmol) to serum proteins was assessed using an ultrafiltration assay as previously reported (Supplementary Material) [13].

### Cell culture

Human embryonic kidney (HEK) cells transfected with human ACE, herein referred to as HEK-ACE cells, and HEK cells transfected with human ACE2, herein referred to as HEK-ACE2 cells, were obtained from Innoprot (Innovative Technologies in Biological Systems S.L. Bizkaia, Spain). These transfected HEK cells were cultured in Dulbecco’s Modified Eagle Medium (DMEM) supplemented with non-essential amino acids, fetal calf serum, antibiotics and hygromycin B as previously reported [13].

### Determination of cell uptake and relative ACE-binding affinity of the radioconjugates

The cell uptake and internalization of the radiopeptides (20 MBq/nmol) were determined using HEK-ACE and HEK-ACE2 cells as previously reported (Supplementary Material) [13]. Statistical analysis was performed by applying a paired t-test to characterize the effect of the linker length, the type of chelator and employed radiometal, respectively, on the radioconjugates’ cell uptake (GraphPad Prism software, version 8.3.1).

The ACE-binding affinity was determined for the ^67^Ga/^nat^Ga-labeled lisinopril conjugates (5 MBq/nmol) using HEK-ACE cells in suspension. In brief, increasing concentrations (1‒2000 nM) of the lisinopril metal conjugates were incubated with 2.5 × 10^5^ HEK-ACE cells at 37 °C for 1 h. Some of the cell samples were co-incubated with an excess of lisinopril (20 µM) to determine the unspecific binding of the lisinopril metal conjugates. After centrifugation and several washing steps, the cells were lysed and the associated activity determined using a γ-counter (Wallac Wizard 1480, PerkinElmer). The K_D_ values were obtained by applying a non-linear regression to the values for specific binding (obtained by subtracting non-specific binding from the total binding). The ACE-binding affinities of the radioconjugates were reported as inverse K_D_ values relative to that of [^67^Ga]Ga-DOTA-LIS-02 which was set as 1.0 and presented as the average ± SD of *n* = 3 independent experiments (Supplementary Material).

### Ethical approval of the in vivo studies and mouse model

All applicable international, national, and/or institutional guidelines for the care and use of laboratory animals were followed and the animal experiments were carried out according to the guidelines of the Swiss Regulations for Animal Welfare. Preclinical studies were ethically approved by the Cantonal Committee of Animal Experimentation and permitted by the responsible cantonal authorities (License N° 75743). Five-week-old female Crl: CD1-*Foxn*^*nu*^ (CD1/nude) mice were obtained from Charles River Laboratories (Sulzfeld, Germany) and fed with standard rodent chow ad libitum. The mice were subcutaneously inoculated with HEK-ACE (8 × 10^6^ cells in 100 µL PBS) on the left shoulder and approximately one week later with HEK-ACE2 cells (4–6 × 10^6^ cells in 100 µL PBS) on the right shoulder. Imaging and biodistribution studies were performed 2‒4 weeks later when the xenografts reached a size of 100–300 mm^3^.

### Biodistribution studies in xenografted mice

Mice were intravenously injected with 3 MBq [^67^Ga]Ga-DOTA-LIS-02 or 3 MBq [^67^Ga]Ga-NODAGA-LIS-02 (0.5 nmol, 100 µL) or 3 MBq [^44^Sc]Sc-DOTA-LIS-02 (1 nmol, 100 µL) diluted in saline containing 0.05% bovine serum albumin (BSA). The mice were sacrificed 1 h and 3 h after administration of the respective radioconjugate. Selected tissues and organs were collected, weighed and counted for activity using the γ-counter. The results were listed as a percentage of the injected activity per gram of tissue mass (% IA/g), using counts of a defined volume of the original injection solution measured at the same time to calculate decay-corrected values. Statistical analysis was performed by applying a regular one-way ANOVA test with a Tukey’s multiple comparison post‐hoc test to compare the data of the radioconjugates. A *p*-value of < 0.05 was considered as statistically significant (GraphPad Prism software, version 8.3.1).

### SPECT/CT and PET/CT imaging studies

SPECT/CT imaging was performed using a four-head, multiplexing, multipinhole small-animal SPECT/CT camera (NanoSPECT/CT™, Mediso Medical Imaging Systems, Budapest, Hungary). SPECT scans were performed at 1 h, 3 h and 24 h after injection of 10 MBq [^67^Ga]Ga-DOTA-LIS-02 or 10 MBq [^67^Ga]Ga-NODAGA-LIS-02 (0.5 nmol), diluted in 100 µL saline containing 0.05% BSA (Supplementary Material). PET/CT scans were performed using a small-animal PET/CT scanner (G8 PET/CT; Xodus Imaging CA, USA [24]). Static whole-body PET scans were performed at 1 h and 3 h after intravenous injection of 3 MBq [^44^Sc]Sc-DOTA-LIS-02 (1 nmol) diluted in 100 µL saline containing 0.05% BSA (Supplementary Material). All images were prepared using the VivoQuant post-processing software (version 3.5, inviCRO Imaging Services and Software, Boston, USA). A Gauss post-reconstruction filter (full width at half maximum, 1 mm) was applied. The scale of activity for the SPECT scans was set to 10 Bq/voxel indicated as 100% with 1 Bq/voxel (10%) cut from the lower scale. The PET scanner was not calibrated for scandium-44, hence, the scale of activity set as 100% with 1% cut from the lower scale.

### In vitro autoradiography studies using [^67^Ga]Ga-DOTA-LIS-02

In vitro autoradiography studies were performed with [^67^Ga]Ga-DOTA-LIS-02 (80 MBq/nmol) on 20-µm thick frozen tissue sections of HEK-ACE and HEK-ACE2 xenografts as well as sections of the kidneys and lungs using a previously reported protocol (Supplementary Material) [25]. The sections were incubated in a Tris-HCl buffer with 0.25% BSA for 10 min before exposure to [^67^Ga]Ga-DOTA-LIS-02 (225 kBq/150 µL) in Tris-HCl buffer with 1% BSA for 60 min at room temperature without or with excess lisinopril (10 µM). After several rinsing steps, the sections were dried and exposed to a phosphor screen (Super resolution screen PSR10450013, PerkinElmer), which was read using a storage phosphor imager (Cyclone Plus, PerkinElmer). The signals were quantified using OptiQuant software (version 5.0, Bright Instrument Co Ltd., PerkinElmer). The average signal intensity on tissue incubated with excess lisinopril was expressed as percentage of the signal on the tissue incubated with the radioconjugate only (set as 100%). Data were obtained from two independent experiments on normal organs and xenografts of *n* = 2‒3 mice.

### Immunohistochemistry studies on mouse paraffin tissue

Immunohistochemical staining of ACE was performed with an anti-ACE antibody (EPR2757; 1:200, Abcam Cambridge, UK) on 2-µm thick paraffin sections of HEK-ACE and HEK-ACE2 xenografts as well as sections of the kidneys and the lungs (Supplementary Material).

### Ex vivo autoradiography study of tissue from mice injected with [^67^Ga]Ga-DOTA-LIS-02

CD1/nude mice without xenografts were injected with [^67^Ga]Ga-DOTA-LIS-02 (25 MBq; 0.3 nmol) in 100 µL saline with 0.05% BSA without or with addition of lisinopril (10 nmol/mouse). The mice were sacrificed at 1–3 h p.i. followed by sequential perfusion with PBS, *p*-formaldehyde solution (4%) and a mixture of PBS and Tissue-Tek O.C.T. (1:1, *v/v*). The lungs and kidneys were collected, embedded in Tissue-Tek O.C.T. and frozen at − 80 °C. Sections of 20-µm thickness were prepared using a cryostat (Epredia Cryostar NX70 Cryostat, Microm International GmbH, Histocom, Zug, Switzerland). Images of the sections were obtained as described for in vitro autoradiograms. The average intensity of the signal present in tissue of *n* = 2 mice that received excess lisinopril was expressed as percentage of the average signal obtained from tissue of *n* = 2 mice injected with the radioconjugate only (set as 100%).

### Molecular docking and dynamics simulations on lisinopril conjugates

Computational studies were performed to propose plausible binding modes of Ga-DOTA-LIS-01 and Ga-DOTA-LIS-02 to human ACE based on the previously reported crystal structure of the ACE/lisinopril complex derived from the Protein Data Bank (PDB; PDB-ID 1O86). The structures of Ga-DOTA-LIS-01 and Ga-DOTA-LIS-02 were prepared with Avogadro software (version 1.2.0) [26] while the ACE protein structure was refined using the UCSF Chimera software (version 1.17.3) [27]. Docking simulations were conducted using the AutoDock Vina software (version 1.2.3) [28] while molecular dynamic simulations were performed using Gromacs (version 2021.1) [29, 30] under the Charmm36 force field. The parameters for Ga-DOTA-LIS-01 and Ga-DOTA-LIS-02 were obtained through the CGenFF website (Supplementary Material) [31].

## Results

### Synthesis of the lisinopril-based conjugates

The esterification of the carboxylic functionalities of lisinopril was readily achieved with an overall yield of 91% in a single synthetic step without requiring any further purification. The lisinopril diethyl ester was used for the synthesis of DOTA-LIS-01, DOTA-LIS-02 and NODAGA-LIS-02, obtained in 9 synthetic steps with an overall yield of 12‒26% and a chemical purity > 98% as determined by analytical HPLC (Supplementary Material, Fig. S1). The chemical identity of the conjugates was confirmed by HRMS analysis (Supplementary Material, Figs. S2-S4, Table S1).

### Radiolabeling and stability of the lisinopril radioconjugates

**Fig. 1:**
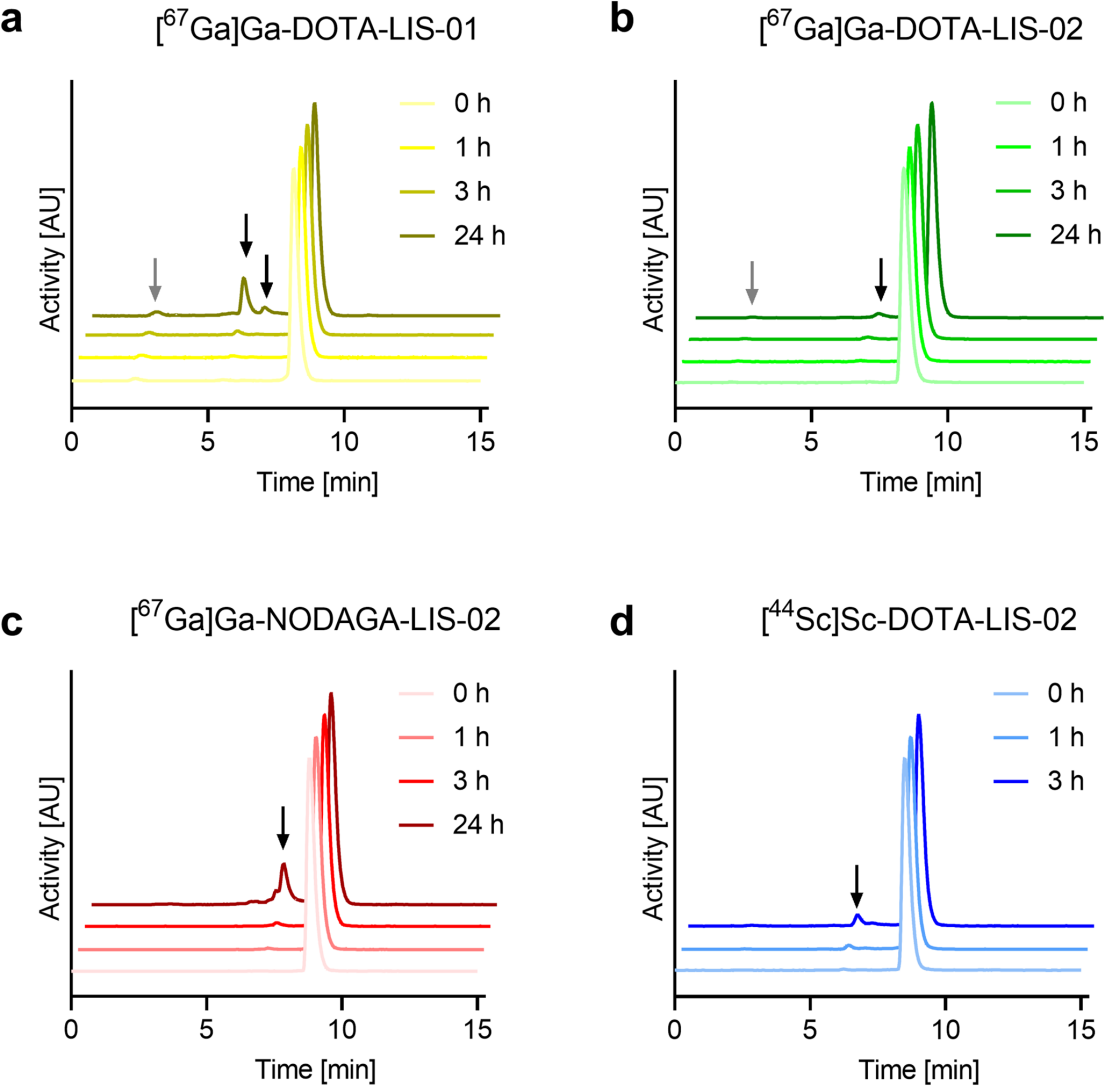
**a-c** HPLC chromatograms of the radioconjugates investigated for radiolytic stability over 24 h. (**a**) [^67^Ga]Ga-DOTA-LIS-01; (**b**) [^67^Ga]Ga-DOTA-LIS-02 and (**c**) [^67^Ga]Ga-NODAGA-LIS-02. **d** HPLC chromatograms of [^44^Sc]Sc-DOTA-LIS-02 investigated for radiolytic stability over 3 h. Gray arrows = free/released radionuclide; black arrows = degradation products of unknown structure

DOTA-LIS-01, DOTA-LIS-02 and NODAGA-LIS-02 radiolabeled with gallium-67 at molar activities of up to 80 MBq/nmol were obtained with > 98% radiochemical purity. Radiolabeling of DOTA-LIS-02 with scandium-44 was feasible at up to 20 MBq/nmol with > 98% radiochemical purity. [^67^Ga]Ga-DOTA-LIS-01, [^67^Ga]Ga-DOTA-LIS-02, [^67^Ga]Ga-NODAGA-LIS-02 and [^44^Sc]Sc-DOTA-LIS-02 were completely stable with > 98% intact radioconjugate found after a 3-h incubation period in saline, even at the highest activity concentration used for in vitro and in vivo experiments. [^67^Ga]Ga-DOTA-LIS-01 and [^67^Ga]Ga-NODAGA-LIS-02 showed partial radiolysis demonstrated by 89 ± 7% and 73 ± 2% intact radioconjugate, respectively, after a 24-h incubation period. No significant radiolytic degradation was, however, observed for [^67^Ga]Ga-DOTA-LIS-02 with ≥ 98% intact radioconjugate detected after the same incubation period. [^44^Sc]Sc-DOTA-LIS-02 was investigated over 3 h due to its shorter half-life. During this period, the radioconjugate remained almost completely stable (Fig. 1; Supplementary Material, Table S2).

In kidney and liver homogenates as well as mouse and human blood plasma, [^67^Ga]Ga-DOTA-LIS-02 was stable over 1 h. Release of ~ 6% gallium-67 from the chelator was observed after a 3-h incubation period in both human and mouse plasma, however. In contrast, [^67^Ga]Ga-NODAGA-LIS-02 was completely stable under all test conditions for the entire duration of the experiments (Supplementary Material, Table S3).

### Distribution coefficient and serum protein-binding properties of the radioconjugates

The *n*-octanol/PBS distribution coefficients of the lisinopril radioconjugates were generally low, indicating a hydrophilic character. A logD value of − 5.47 ± 0.17 was determined for [^67^Ga]Ga-DOTA-LIS-01 while the value for [^67^Ga]Ga-DOTA-LIS-02 was − 5.15 ± 0.13. The exchange of DOTA by NODAGA in [^67^Ga]Ga-NODAGA-LIS-02 resulted in a slighlty higher logD value of − 5.04 ± 0.09. [^44^Sc]Sc-DOTA-LIS-02 showed a logD value of − 4.49 ± 0.01, which can be ascribed to the coordination chemistry of scandium that is different than for gallium (Supplementary Material, Table S4). The lisinopril radioconjugates showed weak binding to mouse and human serum proteins. The binding affinity was similar among all tested radioconjugates and did not drastically differ between mouse and human serum proteins (Supplementary Material, Table S5).

### HEK-ACE and HEK-ACE2 cell uptake and binding affinity of the radioconjugates

**Fig. 2:**
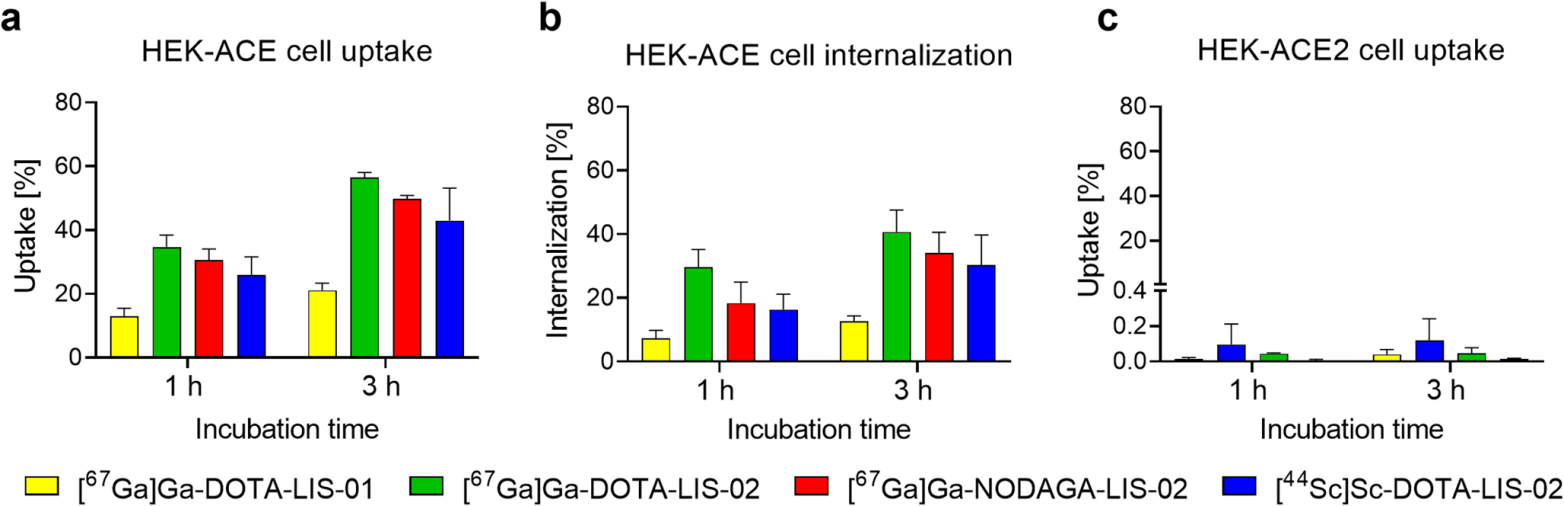
**a-c** Cell uptake and internalization of [^67^Ga]Ga-DOTA-LIS-01, [^67^Ga]Ga-DOTA-LIS-02, [^67^Ga]Ga-NODAGA-LIS-02 and [^44^Sc]Sc-DOTA-LIS-02 after 1 h and 3 h incubation. (**a**) Uptake (surface-bound and internalized fraction) of the radioconjugates in HEK-ACE cells; (**b**) Cell internalized fraction of the radioconjugates in HEK-ACE cells; (**c**) Uptake of the radioconjugates in HEK-ACE2 cells

The uptake of [^67^Ga]Ga-DOTA-LIS-01 in HEK-ACE cells reached 13 ± 3% after 1 h and 21 ± 2% after 3 h incubation. The cell uptake of [^67^Ga]Ga-DOTA-LIS-02 and [^67^Ga]Ga-NODAGA-LIS-02 was significantly higher reaching 31‒35% after 1 h and 50‒57% after a 3-h incubation period (*p* < 0.05, Fig. 2a). In all cases, almost the entire receptor-bound activity was found internalized already after a 1-h incubation period (Fig. 2b). The cell uptake and internalization of [^44^Sc]Sc-DOTA-LIS-02 reached 25 ± 5% and 40 ± 10% after 1 h and 3 h incubation, respectively, which was in the same range as for the ^67^Ga-labeled counterpart (*p* > 0.05). The uptake of all lisinopril radioconjugates into HEK-ACE2 cells was < 0.2% at both investigated timepoints (Fig. 2c). The ACE-binding affinity of [^67^Ga]Ga-DOTA-LIS-02 was 1.8-fold higher than for [^67^Ga]Ga-NODAGA-LIS-02 and 2.4-fold higher than for [^67^Ga]Ga-DOTA-LIS-01 (Supplementary Material, Table S6).

### Biodistribution of ^67^Ga- and ^44^Sc-labeled radioconjugates in xenografted mice

**Fig. 3:**
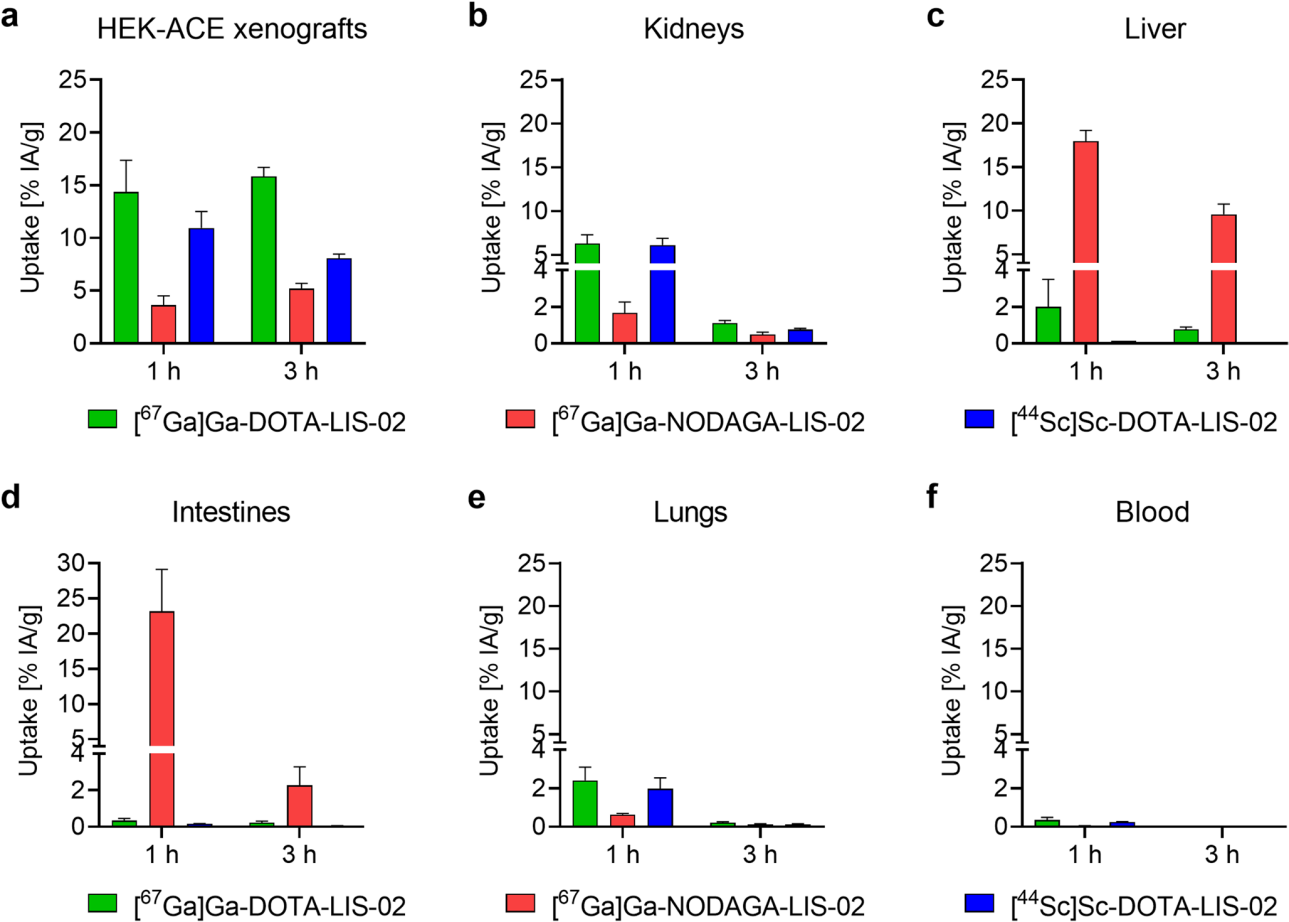
**a-f** Biodistribution data obtained at 1 h and 3 h after injection of [^67^Ga]Ga-DOTA-LIS-02, [^67^Ga]Ga-NODAGA-LIS-02 and [^44^Sc]Sc-DOTA-LIS-02 in HEK-ACE2/ACE xenograft-bearing nude mice. (**a**) HEK-ACE xenograft; (**b**) Kidneys; (**c**) Liver; (**d**) Intestines; (**e**) Lungs and (**f**) Blood. The values represent the average ± SD of *n* = 3 mice

[^67^Ga]Ga-DOTA-LIS-02 reached 14 ± 3% IA/g and 16 ± 1% IA/g uptake in HEK-ACE xenografts at 1 h and 3 h p.i., respectively, while the uptake at these same timepoints was only 3.6 ± 0.9% IA/g at 1 h and 5.2 ± 0.5% IA/g at 3 h after injection of [^67^Ga]Ga-NODAGA-LIS-02 (Fig. 3a; Supplementary material, Table S7). Both radioconjugates showed negligible uptake in HEK-ACE2 xenografts (< 0.3% IA/g). Kidney uptake of [^67^Ga]Ga-DOTA-LIS-02 was 6.3 ± 1.0% IA/g at 1 h p.i. and dropped to 1.1 ± 0.2% IA/g at 3 h p.i. In contrast, kidney retention of [^67^Ga]Ga-NODAGA-LIS-02 was only 1.7 ± 0.6% IA/g at 1 h p.i. and 0.49 ± 0.13% IA/g at 3 h p.i. (Fig. 3b). Liver accumulation of [^67^Ga]Ga-NODAGA-LIS-02 was 18 ± 1% IA/g at 1 h p.i. and 9.6 ± 1.2% IA/g at 3 h p.i., while in the case of [^67^Ga]Ga-DOTA-LIS-02, the uptake in the liver was negligible (≤ 2.0% IA/g already at 1 h p.i.) (Fig. 3c). [^67^Ga]Ga-NODAGA-LIS-02 accumulated in the intestinal tract with 23 ± 6% IA/g at 1 h p.i. while the retention of [^67^Ga]Ga-DOTA-LIS-02 was negligible (< 0.4% IA/g) (Fig. 3d). [^67^Ga]Ga-DOTA-LIS-02 accumulated in the lungs (2.4 ± 0.7% IA/g at 1 h p.i.), while the uptake of [^67^Ga]Ga-NODAGA-LIS-02 in this organ was only 0.63 ± 0.06 IA/g at 1 h (Fig. 3e). [^67^Ga]Ga-DOTA-LIS-02 and [^67^Ga]Ga-NODAGA-LIS-02 were rapidly cleared from the blood circulation resulting in < 0.5% IA/g in the blood pool at 1 h p.i. (Fig. 3f). Overall, the exchange of gallium-67 with scandium-44 to obtain [^44^Sc]Sc-DOTA-LIS-02 did not substantially change the tissue distribution profile. Uptake in the HEK-ACE xenograft reached 11 ± 2% IA/g at 1 h and 8.1 ± 0.4% IA/g at 3 h after injection of [^44^Sc]Sc-DOTA-LIS-02.

### Nuclear imaging after injection of lisinopril radioconjugates in xenografted mice

**Fig. 4:**
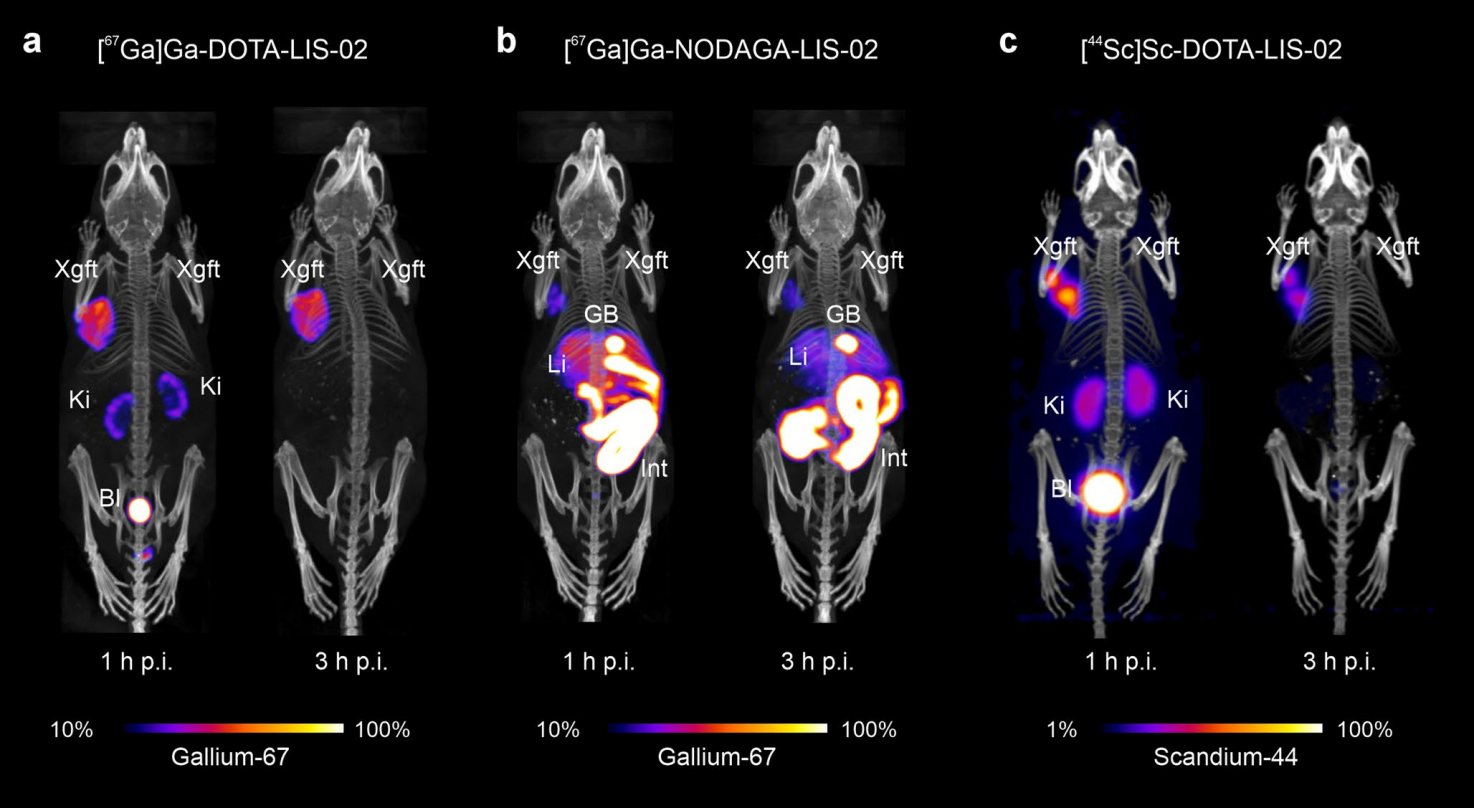
**a/b** SPECT/CT images of HEK-ACE and HEK-ACE2 xenograft-bearing mice acquired at 1 h and 3 h after injection of the ^67^Ga-labeled lisinopril radioconjugates. **c** PET/CT images of HEK-ACE and HEK-ACE2 xenografted mice acquired at 1 h and 3 h after injection of [^44^Sc]Sc-DOTA-LIS-02. (**a**) Mouse injected with [^67^Ga]Ga-DOTA-LIS-02; (**b**) Mouse injected with [^67^Ga]Ga-NODAGA-LIS-02; (**c**) Mouse injected with [^44^Sc]Sc-DOTA-LIS-02

SPECT/CT images of mice injected with the lisinopril radioconjugates visualized the whole-body tissue distribution profiles. [^67^Ga]Ga-DOTA-LIS-02 showed high accumulation in the HEK-ACE xenograft at 1 h p.i., which was retained until 3 h p.i. Kidney accumulation was visible at 1 h p.i. but cleared completely over the following 2 h (Fig. 4a/b). In contrast, the uptake of [^67^Ga]Ga-NODAGA-LIS-02 in the HEK-ACE-xenograft was lower compared to that of [^67^Ga]Ga-DOTA-LIS-02 and substantial accumulation in the liver, gall bladder and intestines was seen already at 1 h p.i. At 3 h p.i., the liver uptake decreased but the signal in the gall bladder and intestines remained high (Fig. 4b). PET/CT images obtained with [^44^Sc]Sc-DOTA-LIS-02 resulted in similar outcome as observed for [^67^Ga]Ga-DOTA-LIS-02 (Fig. 4c). None of the radioconjugates accumulated in the HEK-ACE2 xenograft (Fig. 4).

### ACE expression in xenografts and physiological mouse tissue

**Fig. 5:**
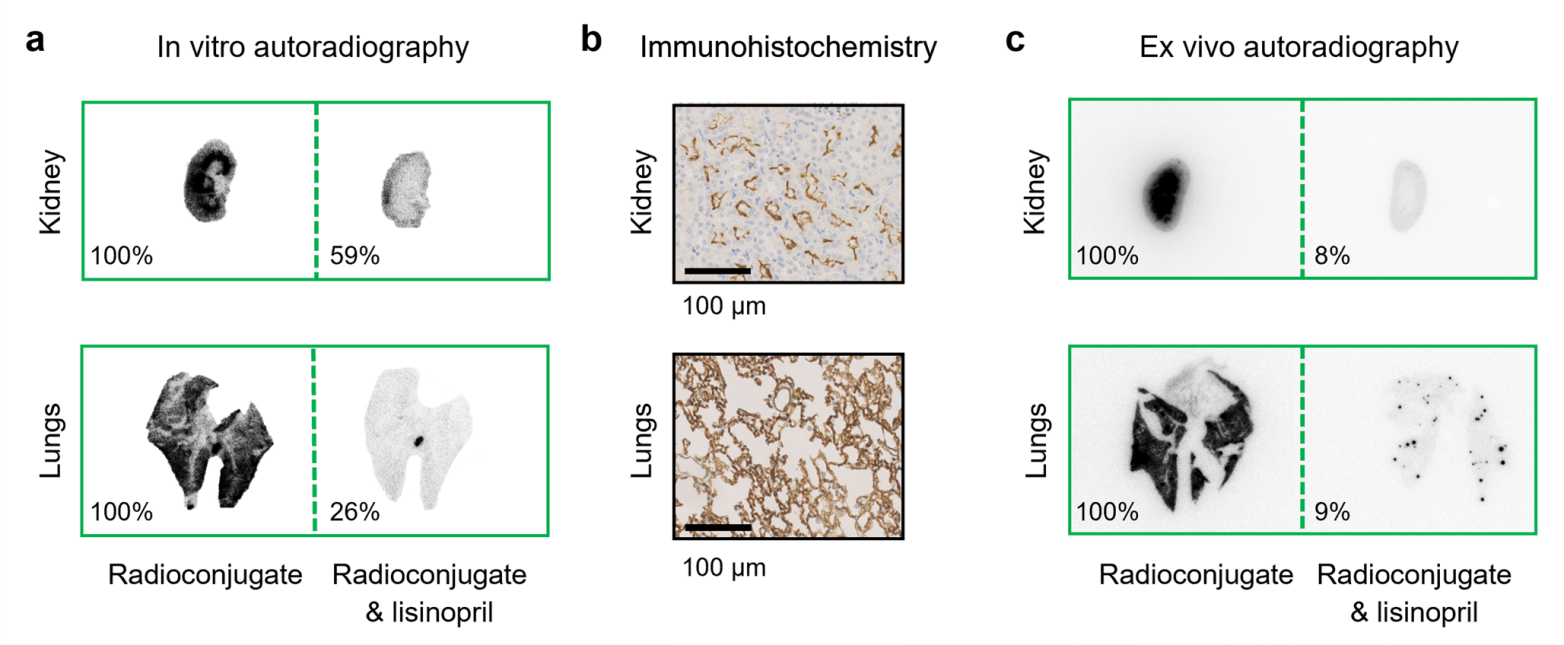
**a** Representative in vitro autoradiograms of mouse kidney and lung sections incubated with [^67^Ga]Ga-DOTA-LIS-02 in absence or presence of excess lisinopril. **b** Representative immunohistochemical ACE staining of mouse kidney and lung tissue. **c** Representative ex vivo autoradiograms of kidney and lung tissue collected from mice 1 h after injection of [^67^Ga]Ga-DOTA-LIS-02 only or co-injected with 10 nmol lisinopril to block ACE. The signal intensity in the tissue of mice co-injected with lisinopril is expressed as the percentage of the signal obtained in tissues of mice injected with the radioconjugate only (average from *n* = 2 mice)

The analysis of the in vitro autoradiograms of HEK-ACE and HEK-ACE2 xenograft tissue sections confirmed specific binding of [^67^Ga]Ga-DOTA-LIS-02 to ACE, but only a negligible signal was observed on tissue sections expressing ACE2. The ACE-specific binding of [^67^Ga]Ga-DOTA-LIS-02 was also observed in lung and kidney tissue sections (Fig. 5a). Quantification of the activity per tissue area revealed the highest radioconjugate binding in the lungs among all investigated physiological mouse tissues. However, the signal obtained for HEK-ACE xenograft tissue was about 50-fold more intense (Supplementary material, Fig. S5a). The receptor expression in xenografts and physiological mouse tissue was confirmed by immunohistochemical staining of paraffin tissue sections (Fig. 5b; Supplementary material, Fig. S5b).

The ex vivo autoradiography of the lungs from immunocompetent mice collected 1 h after injection of [^67^Ga]Ga-DOTA-LIS-02 showed a clear signal which was reduced to 9% in lungs obtained from mice co-injected with excess lisinopril to block the ACE specific binding. The signal of lung tissue collected 3 h after injection of the radioconjugate was still detectable, but the reduction in signal intensity after injection of excess lisinopril was less pronounced (data not shown). At the 1-h p.i. timepoint, a high signal was also detected from kidney sections of mice injected with the radioconjugate only, while only a weak signal of 8% was found in the kidneys of mice co-injected with lisinopril (Fig. 5c). At the 3-h p.i. timepoint, no signal was detected in kidney tissue, presumably due to the efficient excretion of the radioconjugate.

### Molecular docking and dynamics simulations of lisinopril conjugates

**Fig. 6:**
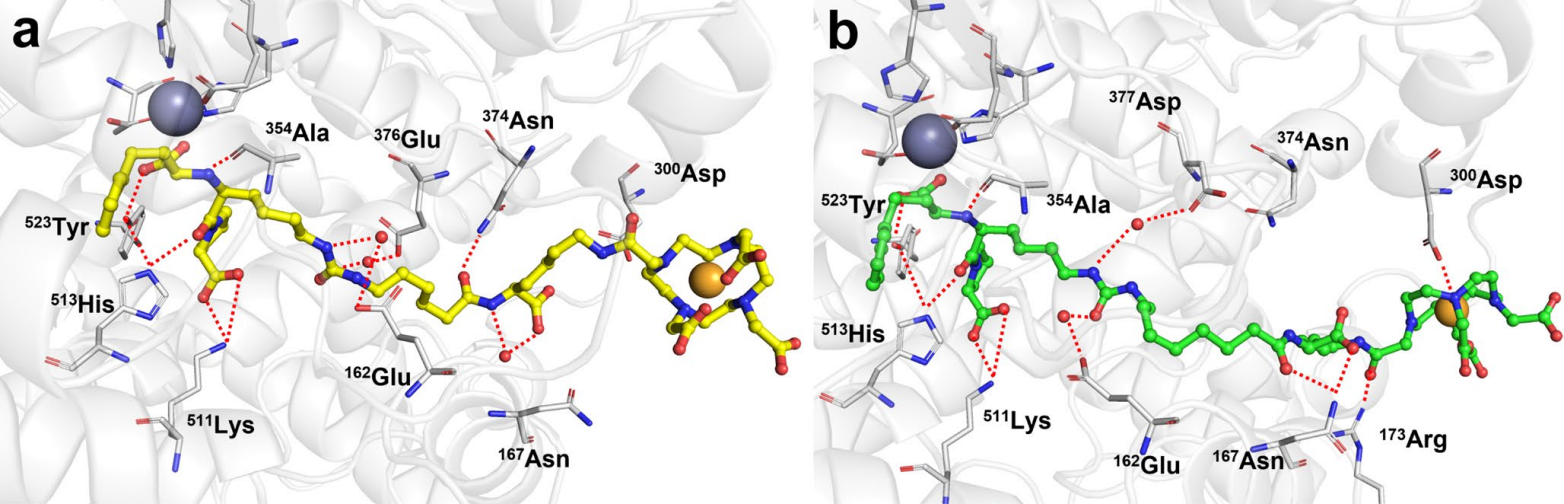
**a** Interactions between ACE, water molecules and Ga-DOTA-LIS-01 (yellow) extracted from the last 25 ns of unbiased Molecular Dynamics (snapshot at 85 ns). **b** Interactions between ACE, water molecules and Ga-DOTA-LIS-02 (green) extracted from the last 25 ns of unbiased molecular dynamics (snapshot at 85 ns). Zinc and gallium are depicted as grey and gold spheres, respectively. Red dashed lines indicate hydrogen bonds or electrostatic interactions

Stable and reliable complexes of ACE with Ga-DOTA-LIS-01 and Ga-DOTA-LIS-02 were obtained after about 50 ns of unbiased molecular dynamics simulations (Fig. 6; Supplementary material, Fig. S6) performed on a model of ACE’s C-domain. In both molecular complexes, the lisinopril moieties interacted through a network of hydrogen bonds with ^354^Ala, ^511^Lys, ^513^His and ^523^Tyr. The ureido functionality of Ga-DOTA-LIS-01 and Ga-DOTA-LIS-02 was also engaged in water mediated interactions with the sidechains of ^162^Glu and ^376^Glu or ^377^Asp, respectively, of ACE. The main differences in the two binding modes were instead identified at the linker and DOTA chelator. Compared to unmodified lisinopril, the Ga-DOTA-LIS-01 interaction with ACE was stabilized only through an additional hydrogen bond with ^374^Asn. The longer linker of Ga-DOTA-LIS-02 allowed the compound to establish two hydrogen bonds with ^167^Asn and ^173^Arg residues of ACE, as well as a strong electrostatic interaction between the Ga-complex and the ^300^Asp sidechain of ACE. These additional interactions of Ga-DOTA-LIS-02 resulted in a more stable complex (Supplementary material) and a favorable short-range Coulomb interaction (Supplementary material Fig. S6e/f). The protein/ligand interaction energies, computed as the sum of short-range Coulomb and Lennard-Jones potential and without accounting for the water contribution, resulted in − 613 ± 66 kJ/mol for ACE/Ga-DOTA-LIS-01 and − 771 ± 53 kJ/mol, for ACE/Ga-DOTA-LIS-02.

## Discussion

The lisinopril-based radioconjugates for ACE-targeting were prepared using solid-phase peptide synthesis with commercial lisinopril as the starting material. The respective conjugates were prepared efficiently and in high purity, but only moderate overall yields. This was not of concern as minute amounts of these compounds were sufficient to prepare the radioconjugates. All radioconjugates were stable in saline for up to 3 h. [^67^Ga]Ga-DOTA-LIS-02 was even stable for an entire day without additional preservatives.

The uptake of [^67^Ga]Ga-DOTA-LIS-02 and [^67^Ga]Ga-NODAGA-LIS-02 in HEK-ACE cells was similar, while [^67^Ga]Ga-DOTA-LIS-01 showed a 2.4-fold lower uptake. The ACE-mediated cell uptake of the radioconjugates was confirmed in vitro by blocking ACE with excess lisinopril which reduced the cell uptake of the radioconjugates to background levels. Importantly, no uptake of the radioconjugates was observed in HEK-ACE2 cells, confirming their ACE-specific binding further. Based on docking and molecular dynamic simulations, the favorable cell uptake data of [^67^Ga]Ga-DOTA-LIS-02 and [^67^Ga]Ga-NODAGA-LIS-02 can be ascribed to the longer linker between the macrocyclic chelator and lisinopril than is the case in [^67^Ga]Ga-DOTA-LIS-01. This enabled a stronger binding to the ACE cleft. Due to the unfavorable in vitro results obtained with [^67^Ga]Ga-DOTA-LIS-01, this radioconjugate was not further tested in preclinical in vivo studies.

Interestingly, [^67^Ga]Ga-DOTA-LIS-02 and [^67^Ga]Ga-NODAGA-LIS-02 showed differences in the distribution profiles obtained in xenograft-bearing mice, despite the fact that their in vitro properties were quite similar. While [^67^Ga]Ga-DOTA-LIS-02 accumulated mainly in HEK-ACE xenografts and to a minor extent in the kidneys, [^67^Ga]Ga-NODAGA-LIS-02 showed considerably lower accumulation in HEK-ACE xenografts as well as in the kidneys. Instead, [^67^Ga]Ga-NODAGA-LIS-02 was characterized with substantial activity retention in the liver and intestinal tract. The question as to whether these findings were a result of a hepatic metabolization of [^67^Ga]Ga-NODAGA-LIS-02 was addressed by incubating the radioconjugates in a suspension of mouse liver homogenates to test their stability. Interestingly, both [^67^Ga]Ga-DOTA-LIS-02 and [^67^Ga]Ga-NODAGA-LIS-02 remained intact under these conditions over the 3-h investigation period. These data indicate that hepatic metabolism was most probably not the reason for the observed liver accumulation of activity after injection of [^67^Ga]Ga-NODAGA-LIS-02. Rather, the difference in the metal-chelate may be the reason for different affinities to hepatic transporters and related hepatobiliary excretion of the respective radioconjugates. Overall, the favorable target-to-off-target contrast achieved with [^67^Ga]Ga-DOTA-LIS-02 indicates a key advantage of this radioconjugate over [^67^Ga]Ga-NODAGA-LIS-02. While DOTA is not an ideal chelator for gallium-67/68, it may be interesting for a number of other radiometals including scandium-44 [32]. This radionuclide could be of value in the context of these radioconjugates due to its four-fold longer half-life as compared to that of gallium-68 which would enable scanning at later timepoints when target-to-off-target contrast would be improved. Furthermore, a somewhat improved image resolution can be expected for scandium-44 as previously demonstrated in phantom studies and first-in-human application of this novel PET nuclide [33, 34]. [^44^Sc]Sc-DOTA-LIS-02 and [^67^Ga]Ga-DOTA-LIS-02 showed comparable tissue distribution profiles and elimination routes in mice. Small variations in the accumulation in HEK-ACE xenografts and lungs in biodistribution studies, may be attributed to the use of [^44^Sc]Sc-DOTA-LIS-02 at a two-fold lower molar activity compared to that of [^67^Ga]Ga-DOTA-LIS-02 and, hence, double the molar amount of conjugate that was injected.

The documented cross-reactivity of lisinopril among different species [35] led to believe that our radioconjugate would also bind to ACE expressed in normal mouse tissues. Therefore, in vitro autoradiography studies were performed with mouse tissue sections using [^67^Ga]Ga-DOTA-LIS-02 in the absence and presence of lisinopril to detect ACE-specific binding. Indeed, the results correlated well with immunohistochemical stainings of sections from the same organs of mice. Ex vivo autoradiography, referring to investigations of tissue sections of mice that were injected with [^67^Ga]Ga-DOTA-LIS-02 in the absence or presence of excess lisinopril, confirmed the ACE-specific binding of [^67^Ga]Ga-DOTA-LIS-02 in normal lungs and kidney tissue.

[^67^Ga]Ga-DOTA-LIS-02 or [^44^Sc]Sc-DOTA-LIS-02 could, thus, be employed to monitor the expression of ACE in mouse models of various cardiovascular conditions such as abdominal aortic aneurisms, which can be induced through angiotensin-II [36, 37] and, therewith, serve as a valuable research tool. Since the role of RAAS in aneurismal disease remains controversial, using the lisinopril-based radioconjugates ‒ potentially in combination with ACE2-targeting radioconjugates ‒ could improve the understanding of disease mechanisms [38]. In addition, these radioconjugates could be of value for a better understanding of disease progression of atherosclerosis and hypertension using the respective mouse models [39, 40] and, thereby, support medical and scientific communities in developing more effective therapeutic strategies.

A potential limitation of this study refers to the animal model which does not reflect the situation of a disease in which ACE expression may change under (patho)physiological conditions. To more precisely assess the imaging potential of the developed radioconjugates in view of a clinical translation, further preclinical studies involving adequate disease models would be necessary.

## Conclusion

Novel ACE-targeting radioconjugates were designed and investigated preclinically in view of using them as imaging tools of ACE in patients. It was revealed that a longer linker entity between the lisinopril and the chelator was beneficial to achieve a high affinity to ACE and conjugates with a DOTA chelator resulted in a more favorable tissue distribution profile than the one with a NODAGA chelator. DOTA-LIS-02 emerged as the most promising candidate among the different lisinopril-based conjugates, which could be useful for imaging physiological levels of ACE in cardiovascular disease and Covid-19 after labeling with PET radiometals such as gallium-68 or scandium-44.

## Electronic supplementary material

Below is the link to the electronic supplementary material.

Supplementary Material 1

## Data Availability

The movie of the molecular dynamics study (Video S1) is available on Zenodo platform (DOI: 10.5281/zenodo.15311169). The raw data of the results presented in this study are available on request from the corresponding author.
