# Supplementary material for: Development of lisinopril radioconjugates for nuclear imaging of the angiotensin converting enzyme
Source: Eur J Nucl Med Mol Imaging. 2025 Jun 7;52(13):5030–43. doi: 10.1007/s00259-025-07386-w (PMC12589211; doi:10.1007/s00259-025-07386-w)
Supplement: Supplementary file 1 — Supplementary Material 1 [file 259_2025_7386_MOESM1_ESM.docx]

**SUPPLEMENTARY MATERIAL**

**Development of lisinopril radioconjugates for nuclear imaging of the angiotensin converting enzyme**

Christian Vaccarin^1^, Darja Beyer^1^, Anzhelika Moiseeva^1^, Chiara Favaretto^1,2^, Nicholas P. van der Meulen^1,3^, Benjamin D. Hunkeler^1^, Niels J. Rupp^4,5^, Giovanni Marzaro^6^, Roger Schibli^1,7^, Cristina Müller^1,7^*

*1. Center for Radiopharmaceutical Sciences, PSI Center for Life Sciences, Villigen-PSI, Switzerland*

2. *Nuclear Medicine Department, University Hospital Basel, Basel, Switzerland*

*3. Laboratory of Radiochemistry, PSI Center for Energy and Nuclear Safety, Villigen-PSI, Switzerland*

*4. Department of Pathology and Molecular Pathology, University Hospital Zurich, Zurich, Switzerland*

*5. Faculty of Medicine, University of Zurich, Zurich, Switzerland*

*6. Department of Diagnostics and Public Health, University of Verona, Verona, Italy*

*7. Department of Chemistry and Applied Biosciences, ETH Zurich, Zurich, Switzerland*

**1. Synthesis of DOTA-LIS-01, DOTA-LIS-02 and NODAGA-LIS-02**

**Purpose:** Three lisinopril conjugates with a macrocyclic chelator were designed and synthesized. The molecules are referred to as DOTA-LIS-01, DOTA-LIS-02 and NODAGA-LIS-02.

**Methods:**

***Synthesis of the lisinopril diethyl ester (2).*** SOCl_2_ (733 μL, 10 mmol, 10 equiv.) was added to a suspension of lisinopril dihydrate (**1**, 441 mg, 1.0 mmol, 1.0 equiv.) in absolute ethanol (20 ml) and the resulting solution was refluxed under vigorous stirring overnight. The reaction mixture was monitored using thin layer chromatography (TLC, eluent: CHCl_3_/methanol, 9:1, *v/v*). After cooling, the solvent was removed by evaporation under reduced pressure. The oily residue was re-dissolved in a saturated NaHCO­_3_ solution (40 mL) and subsequently extracted with ethyl acetate (3 x 20 mL). The organic phase was dried over Na_2_SO_4_, filtered and concentrated under reduced pressure. The product **2** was used in the following reaction steps without any further purification.

***Synthesis of DOTA-LIS-01, DOTA-LIS-02 and NODAGA-LIS-02.*** 2-Chlorotrytyl chloride resin (2-CTC, 200 mg, 0.3 mmol) was weighed into a 5 mL filter-containing syringe and swelled in dry dichloromethane (DCM) for 1 h. *N*α-Fmoc-*N*ε-(4-allyloxycarbonyl)-l-lysine (Fmoc-Lys(Alloc)-OH, 163 mg, 0.36 mmol, 1.2 equiv.) and *N,N*-diisopropylethylamine (DIPEA, 430 μL, 2.4 mmol, 8.0 equiv.) were dissolved in dry DCM (3 mL), added to the resin and stirred overnight. Unreacted 2-CTC sites were capped by shaking the resin in a mixture of DCM, methanol and DIPEA (17:2:1, *v/v/v*) for 30 min. After each reaction step, unreacted reagents were removed from the resin by washing with DCM or dimethylformamide (DMF). Subsequently, the Fmoc protecting group was cleaved by shaking the resin in 3 mL of a 1:1 (*v/v*) mixture of DMF and piperidine twice for 5 min. The obtained resin-immobilized intermediate **4** was split into equal portions of 0.10 mmol each. *N*-Fmoc-6-aminohexanoic acid (Fmoc-Ahx-OH, 141 mg, 0.4 mmol, 4.0 equiv.) or *N*-Fmoc-8-aminooctanoic acid (Fmoc-Aoc-OH, 152 mg, 0.4 mmol, 4.0 equiv.) were activated for 2 min with *O*-(benzotriazol-1-yl)-*N,N,N′,N’*-tetramethyluronium-hexafluorophosphate (HBTU, 150 mg, 0.396 mmol, 3.96 equiv.) in the presence of DIPEA (139 μL, 0.8 mmol, 8.0 equiv.) in dry DMF (3 mL) before being added to the resin and agitated for 1 h, after which the Fmoc protecting group was cleaved as described above. In parallel, a solution of DIPEA (2.4 mL) in DCM (2 mL) was added over 1.5 h to an ice-cold solution of lisinopril diethyl ester (**2**, 185 mg, 0.40 mmol, 4.0 equiv.) and triphosgene (39 mg, 0.13 mmol, 1.3 equiv.) in DCM (4 mL) followed by further stirring for 4 h. At this stage, the resin-immobilized compounds **5** or **6**, respectively, were added to the reaction mixture and stirred at room temperature overnight. The resin was collected by filtering through a new filter-containing 5 mL syringe. The *N*ε-Alloc protecting group was cleaved from compounds **7** and **8** within 1 h, using a solution of tetrakis(triphenylphosphine)palladium(0) (35 mg, 0.03 mmol, 0.3 equiv.) and morpholine (260 μL, 3.0 mmol, 30.0 equiv.) in dry DCM (3 mL). Residual palladium traces were removed by washing the resin with 1% DIPEA in DMF (*v/v*) and a sodium diethyldithiocarbamate (15 mg/mL) solution in DMF. *Tert*-butyl 2-(4-(2-(2-aminoethylamino)-2-oxoethyl)-7,10-bis(2-((2-methylpropan-2-yl)oxy)-2-oxoethyl)-1,4,7,10-tetrazacyclododec-1-yl)acetate (DOTA-tri(^t^Bu ester), 259 mg, 0.4 mmol, 4.0 equiv.) or 4-(4,7-bis(2-(*tert*-butoxy)-2-oxoethyl)-1,4,7-triazonan-1-yl)-5-(tert-butoxy)-5-oxopentanoic acid (NODAGA-tri(^t^Bu ester), 218 mg, 0.4 mmol, 4.0 equiv.) was then coupled to the newly formed primary amine group by an HBTU-mediated reaction as described above. Cleavage from the resin and simultaneous removal of acid-labile protecting groups was performed by shaking the resin-immobilized intermediates in a trifluoroacetic (TFA) solution containing 2.5% Milli-Q water and 2.5% triisopropylsilane (*v/v/v*) for 2 h. After removing the volatile solvents by N_2_ stream, the obtained residues were re-dissolved in 4 mL LiOH (1 M) in a 1:1 (*v/v*) mixture of acetonitrile/water and stirred at room temperature for 7 h. After neutralization of the alkaline solution with TFA to obtain pH 7, the desired DOTA-LIS-01, DOTA-LIS-02 and NODAGA-LIS-02 were isolated from the reaction mixture by semipreparative high-performance liquid chromatography (HPLC).

***Purification of DOTA-LIS-01, DOTA-LIS-02 and NODAGA-LIS-02.*** The produced lisinopril conjugates were purified using a Merck-Hitachi LaChrom HPLC system equipped with a D-7000 interface, L-7200 autosampler, L-7400 UV detector, L-7100 pump and a reversed-phase C18 column (C18, Sunfire^TM^, 5 μm, 10×150 mm, Waters, Milford, MA, USA). The products were eluted using a linear gradient of NH_4_HCO_3_ (10 mM) in Milli-Q water (95–80%) and acetonitrile (5–20%) over 15 min at a flow rate of 2.0 mL/min. Fractions containing the pure conjugates were identified by measuring the UV absorbance at λ = 230 nm. The eluate obtained from multiple HPLC runs was combined and collected in a round-bottom flask. The resulting solution was frozen using liquid nitrogen and subjected to lyophilization over at least 24 h. Residual salt traces were removed by re-dissolving the obtained residue in 1 mL Milli-Q water and subsequent re-processing using the same HPLC pump and UV detector as described above, but eluting with a linear gradient of Milli-Q water containing 0.1% (*v/v*) TFA (85–65%) and acetonitrile (15–35%) over 15 min at a flow rate of 2.0 mL/min. A second lyophilization of the product-containing fractions yielded the desired DOTA-LIS-01, DOTA-LIS-02 and NODAGA-LIS-02. The chemical purity of the final lisinopril conjugates was determined by UV/Vis-HPLC analysis (Sunfire^TM^, 5 μm, 4.6×150 mm, Waters, Milford, MA, USA, λ = 254 nm) with a linear gradient of Milli-Q water containing 0.1% TFA (*v/v*) (95–20%) and acetonitrile (5–80%) over 15 min at a flow rate of 1.0 mL/min.

**Results:**

***Chemical characterization of the lisinopril diethyl ester (2).*** Compound **2** was obtained as a colorless oil and used in the next reaction step without further purification. (419 mg, 0.91 mmol, yield: 91%). **^1^H-NMR** (400 MHz, CD_2_Cl_2_): δ[ppm] 7.30 – 7.23 (m, 2H); 7.23 -7.14 (m, 3H); 4.42 (dd, J = 8.9, 4.6 Hz, 1H); 4.18 – 4.09 (m, 4H); 3.67 – 3.47 ( m, 2H); 3.41 - 3.35 (m, 1H); 3.17 (t, J= 6.8 Hz, 1H); 2.88 (t, J= 7.4 Hz, 2H); 2.72 - 2.58 (m, 2H); 2.22 – 2.11 (m, 1H); 2.07 – 1.81 (m, 5H); 1.73 – 1.44 (m, 6H); 1.24 (q, J= 7.16, 6H). **^13^C-NMR** (101 MHz, CD_2_Cl_2_): δ[ppm] 174.95, 173.46, 172.21, 142.17, 129.04, 128.84, 126.44, 61.62, 61.29, 60.19, 59.62, 58.61, 47.30, 40.74, 35.65, 33.07, 32.48, 29.89, 29.46, 25.52, 22.99, 14.68, 15.55. **HRMS** (ESI): calculated for C_25_H_40_N_3_O_5_ [M+H]^+^: 462.2962, found: 462.2955.

***Chemical characterization of DOTA-LIS-01, DOTA-LIS-02 and NODAGA-LIS-02.*** The desired conjugates were obtained as a white powder with a chemical purity of >98%, determined by analytical HPLC (Fig. S1). The chemical identity of the conjugates was confirmed by high-resolution mass spectrometry (HRMS) analysis (Table S1 and Fig. S2-S4).


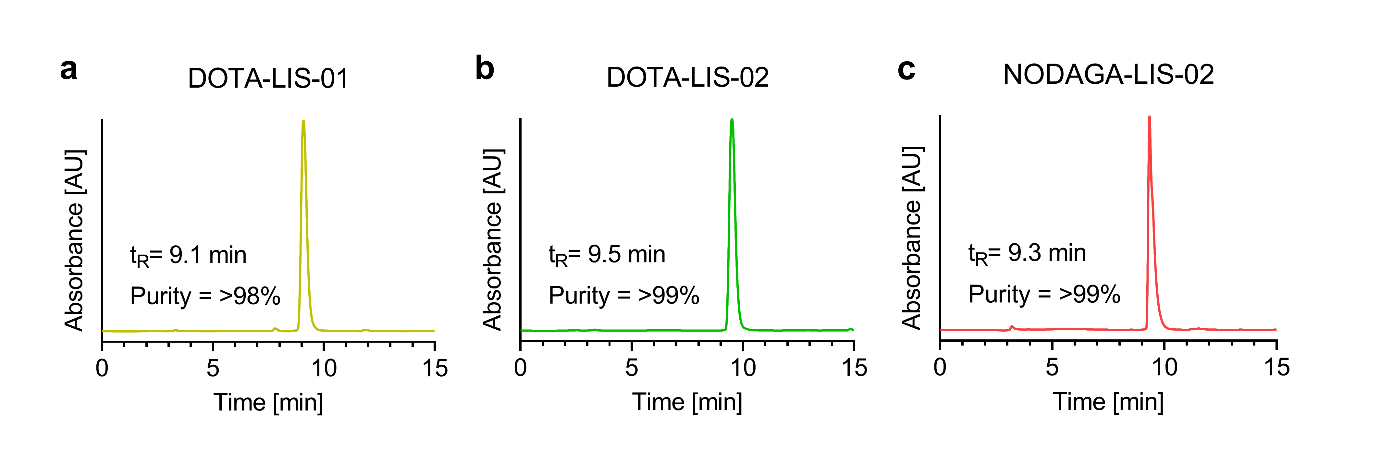
**Fig. S1 a-c** HPLC chromatograms (λ=254 nm) obtained for the quality control of the lisinopril conjugates; (**a**) DOTA-LIS-01, (**b**) DOTA-LIS-02 and (**c**) NODAGA-LIS-02

**Table S1** Data characterizing the synthesized lisinopril conjugates

| **Conjugate** | **Adduct ion** | **m/z_calc_** | **m/z_found_** | **Yield [%]** | **Purity [%]** |
| --- | --- | --- | --- | --- | --- |
| **DOTA-LIS-01** | [M+2H]^2+^ | 539.2950 | 539.2955 | 12% | >98% |
| **DOTA-LIS-02** | [M+2H]^2+^ | 553.3106 | 553.3115 | 24% | >99% |
| **NODAGA-LIS-02** | [M+2H]^2+^ | 538.7973 | 538.7970 | 26% | >99% |


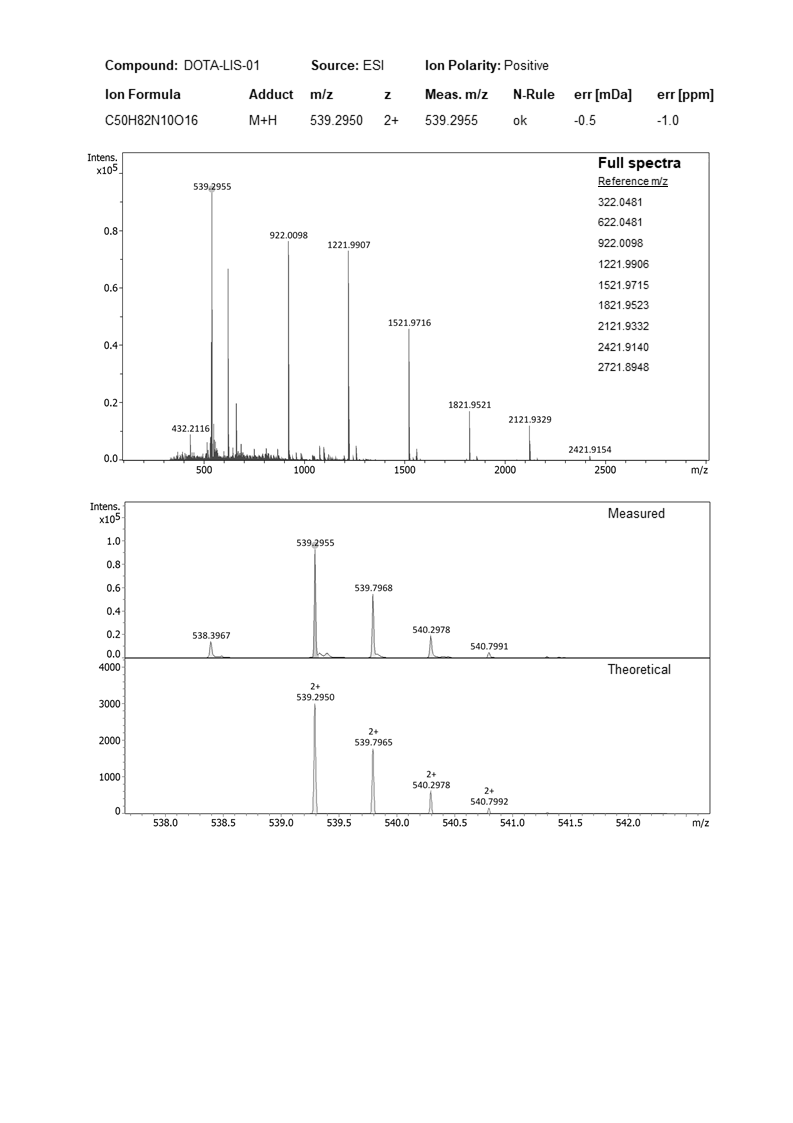


**Fig. S2** Full range HRMS (top) spectra of DOTA-LIS-01 and zoom-in of the comparison between the measured spectra and the theoretical one (bottom)


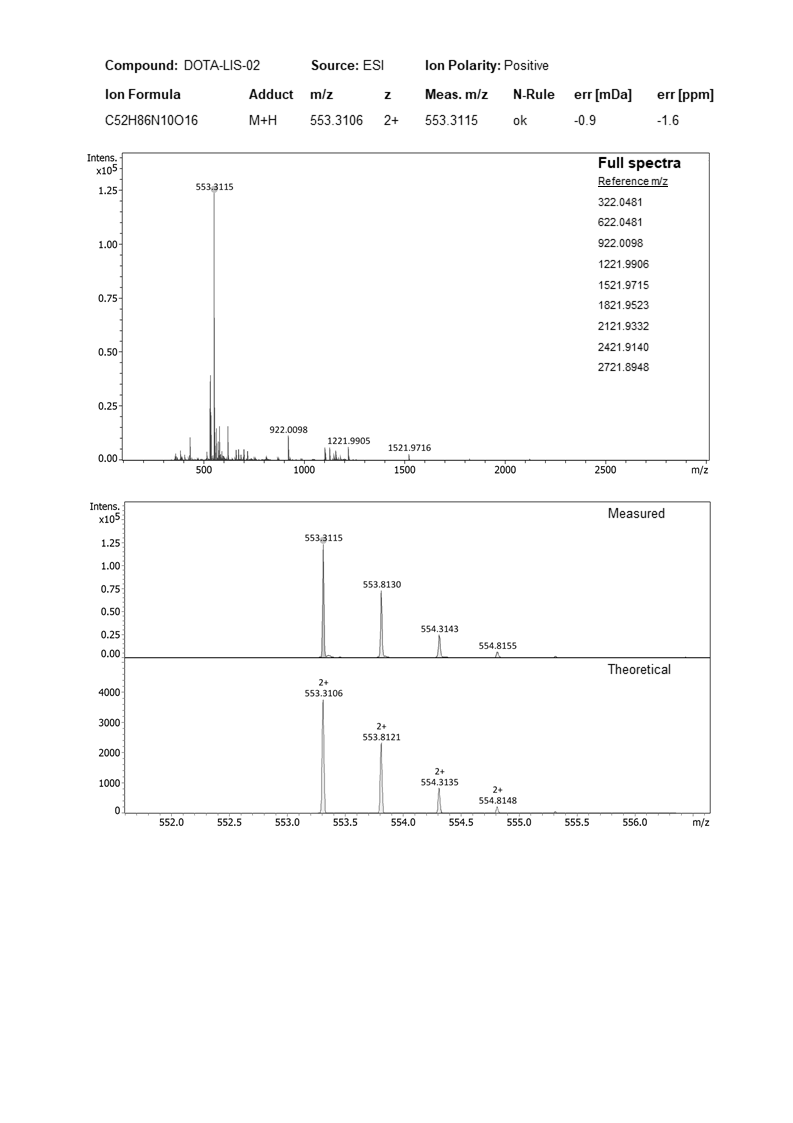


**Fig. S3** Full range HRMS (top) spectra of DOTA-LIS-02 and zoom-in of the comparison between the measured spectra and the theoretical one (bottom)


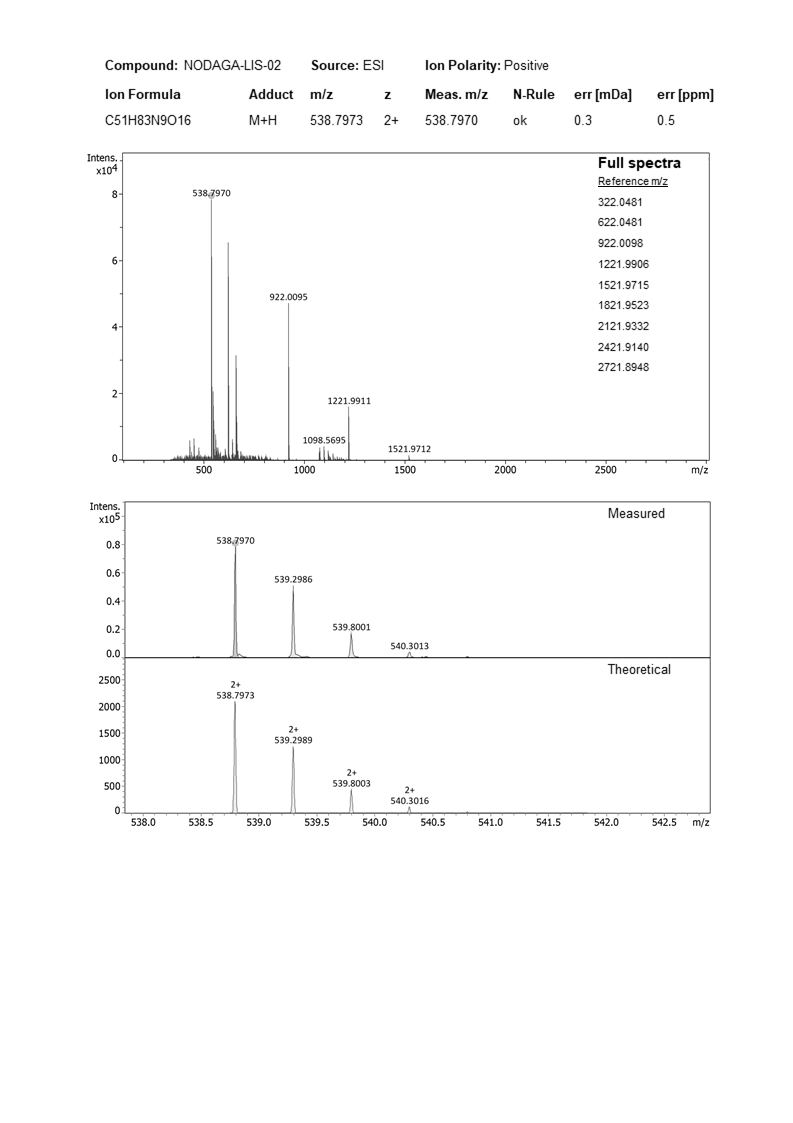


**Fig. S4** Full range HRMS (top) spectra of NODAGA-LIS-02 and zoom-in of the comparison between the measured spectra and the theoretical one (bottom)

**2. Radiolabeling and radiolytic stability of the lisinopril radioconjugates**

**Purpose:** DOTA-LIS-01, DOTA-LIS-02 and NODAGA-LIS-02 were labeled with gallium-67 and, additionally, DOTA-LIS-02 was labeled with scandium-44. The radiolytic stability of the respective lisinopril radioconjugates was investigated over a 24-h period.

**Methods:** [^67^Ga]GaCl_3_ in HCl (0.1 M) was added to a sodium acetate solution (0.5 M) to obtain a buffered system of pH ~4. DOTA-LIS-01, DOTA-LIS-02 or NODAGA-LIS-02 was added from an aqueous stock solution (1 mM) to obtain a defined molar activity (5‒80 MBq/nmol) followed by incubation of the reaction mixture at 95 °C for 10 min. The radiolabeling of DOTA-LIS-02 with scandium-44 is described in the main article. Quality control of the radioconjugates was performed after dilution in Milli-Q water containing sodium diethylenetriamine pentaacetic acid (Na_5_-DTPA; 50 μM) using a Merck Hitachi LaChrom HPLC equipped with a D-7000 interface, a L-7200 autosampler, a radioactivity detector (LB 506 B; Berthold) and a L-7100 pump connected to a C18 column (Xterra^TM^, 5 μm, 4.6×150 mm, Waters, Milford, MA, USA). The radioconjugates were eluted using a linear gradient of acetonitrile (5–80%) and Milli-Q water containing 0.1% (*v/v*) TFA (95–20%) over 15 min at a flow rate of 1.0 mL/min. The radiolytic stability of the radioconjugates (20 MBq/nmol) was investigated at an activity concentration of 10 MBq/100 µL in saline over a period of 24 h by injecting samples of the solution after 1 h, 3 h and 24 h incubation at room temperature. Due to the shorter half-life of scandium-44 (t_1/2_= 4.0 h) the stability of [^44^Sc]Sc-DOTA-LIS-02 was investigated only up to 3 h. The product peak on the chromatogram was expressed as percentage of the sum of integrated peak areas of the entire chromatogram (set as 100%) relative to the value obtained immediately after radiolabeling.

**Results:** The results are reported in the main article and in Table S2.

**Table S2** Radiolytic stability of the lisinopril radioconjugates

| **Radioconjugate** | **Intact radioconjugate [%]** | | |
| --- | --- | --- | --- |
|  | 1 h | 3 h | 24 h |
| [^67^Ga]Ga-DOTA-LIS-01 | 99 ± 1 | 98 ± 1 | 89 ± 7 |
| [^67^Ga]Ga-DOTA-LIS-02 | 99 ± 2 | 99 ± 1 | 98 ± 2 |
| [^67^Ga]Ga-NODAGA-LIS-02 | 99 ± 1 | 99 ± 1 | 73 ± 2 |
| [^44^Sc]Sc-DOTA-LIS-02 | 99 ± 1 | 98 ± 1 | n.d. |

Values are expressed as the average ± standard deviation (SD) of n=3 individually performed experiments; n.d. = not determined

**3. Ex vivo stability of [^67^Ga]Ga-DOTA-LIS-02 and [^67^Ga]Ga-NODAGA-LIS-02**

**Purpose:** The stability of [^67^Ga]Ga-DOTA-LIS-02 and [^67^Ga]Ga-NODAGA-LIS-02 was investigated in blood plasma and samples of organ homogenates.

**Methods:** [^67^Ga]Ga-DOTA-LIS-02 and [^67^Ga]Ga-NODAGA-LIS-02 (6 MBq/nmol) were incubated with the mouse blood plasma (Lot N°: 32321; Rockland Immunochemicals , Inc. PA, USA) and human blood plasma (Blood donation SRK Aargau-Solothurn, Switzerland) as well as with liver and kidney homogenates (5 MBq/200 µL) obtained from CD1/nude mice at 37 °C. The radioconjugates were also incubated in saline as a control. At given time points (30 min, 1 h and 3 h) a 20-µL aliquot was taken and diluted with the same volume of ice-cold NH_4_OH solution (0.025%, *v/v*) in methanol. After centrifugation for 1 min at 2600 rcf at 4 °C, 2 µL (~0.05 MBq) of the supernatant were applied to reversed phase TLC (Silica gel 60 RP-18; Merck). The TLC plates were developed using a mixture (3:7, *v/v*) of acetonitrile and citrate buffer (pH 5.5; 0.1 M) as the mobile phase. The TLC plates were exposed to a phosphor screen (Super resolution screen PSR10450013, PerkinElmer) followed by development using a storage phosphor system (Cyclone Plus, PerkinElmer). The quantification of the signals was carried out using the OptiQuant software (version 5.0, Bright Instrument Co Ltd., PerkinElmer). The chromatograms were analyzed by calculating the peak areas of the intact radioconjugate, the released gallium-67 as well as possible degradation products of unknown structure. The quantity of the intact product was expressed as percentage of the sum of integrated peak areas of the entire chromatogram which was set as 100%.

**Results:** [^67^Ga]Ga-DOTA-LIS-02 and [^67^Ga]Ga-NODAGA-LIS-02 were completely stable after 1 h incubation under any of the reported assay conditions. The results of the 3 h incubation timepoint are reported in the main article and in Table S3.

**Table S3** Stability of [^67^Ga]Ga-DOTA-LIS-02 and [^67^Ga]Ga-NODAGA-LIS-02 in mouse and human blood plasma as well as in mouse liver and kidney homogenates over a period of 3 h

| **Intact compound** | **Saline** | **Mouse plasma** | **Human plasma** | **Liver homogenate** | **Kidney homogenate** |
| --- | --- | --- | --- | --- | --- |
| [^67^Ga]Ga-DOTA-LIS-02 | >99% | 94 ± 1% | 92 ± 2% | >99% | >99% |
| [^67^Ga]Ga-NODAGA-LIS-02 | >99% | >99% | >99% | >99% | >99% |

Values are expressed as the average ± SD of n=3 individually performed experiments; a value of >99% indicates that the radioconjugates remained entirely stable

**4. *n*-Octanol/PBS distribution coefficient**

**Purpose:** The *n*-octanol/PBS distribution coefficient (logD value) was determined to assess the hydrophilic/lipophilic character of the radioconjugates.

**Methods:** A fixed amount of the respective radioconjugate (0.5 MBq, 25 µL) was added to radio-immunoassay (RIA) tubes containing a mixture of PBS pH 7.4 (1475 µL) and *n*-octanol (1500 µL). After vortexing vigorously for 1 min, the tubes were centrifuged (560 rcf) for 6 min to obtain phase separation. The activity in the organic and aqueous phases was determined using a γ-counter (Wallac Wizard 1480, PerkinElmer). The distribution coefficients were expressed as the logarithm of the ratio of counts per minute (cpm) measured in the *n*-octanol phase to the cpm measured in the PBS pH 7.4 phase and indicated as the average of three independent measurements ± SD, each performed with five replicates.

**Results:** The results are reported in the main article and Table S4.

**Table S4** Distribution coefficient measured for the ^67^Ga- and ^44^Sc-labeled lisinopril conjugates

| **Compound** | **logD value** |
| --- | --- |
| [^67^Ga]Ga-DOTA-LIS-01 | −5.47 ± 0.17 |
| [^67^Ga]Ga-DOTA-LIS-02 | −5.15 ± 0.13 |
| [^67^Ga]Ga-NODAGA-LIS-02 | −5.04 ± 0.09 |
| [^44^Sc]Sc-DOTA-LIS-02 | −4.49 ± 0.01 |

Results are shown as average ± SD of n = 3 independent experiments

**5. Blood plasma protein-binding properties**

**Purpose:** [^67^Ga]Ga-DOTA-LIS-01, [^67^Ga]Ga-DOTA-LIS-02, [^67^Ga]Ga-NODAGA-LIS-01 and [^44^Sc]Sc-DOTA-LIS-02 were incubated in mouse and human blood plasma followed by an ultrafiltration assay in order to determine its binding to blood plasma proteins.

**Methods:** The plasma protein binding of the radioconjugates (20 MBq/nmol) was assessed using mouse blood plasma (Lot N°: 32321, Rockland Immunochemicals, Inc., USA,; concentration of mouse serum albumin (MSA): ⁓550 µM) and human blood plasma (Stiftung Blutspende SRK Aargau-Solothurn, Switzerland; concentration of human serum albumin (HSA): ⁓800 µM), respectively. After incubation in mouse and human blood plasma (0.3 MBq in 150 μL) for 30 min at 37 °C, the samples were cooled on ice and diluted with ice-cold PBS (150 μL). The plasma protein-bound fraction was separated from the free (plasma protein-unbound) fraction of each sample by means of Amicon centrifugal filters (Amicon Ultra, 0.5 mL, Merck Millipore; cutoff 10 kDa) by ultrafiltration using a centrifuge (14000 rcf, 30 min at 4 °C). Afterwards, the filter inserts were inverted and centrifuged again (200 rcf) for 3 min to recover the protein-bound fraction of the radioconjugates. The activities of the free radioconjugates in the filtrate, as well as in the filter insert and the plasma protein-bound radioconjugate (bound), were counted for activity separately using a γ-counter (Wallac Wizard 1480, PerkinElmer). The total activity (total) was determined as the sum of the activities in the filtrate, filter insert, and protein-bound fractions. The percentage of radioconjugate bound to MSA and HSA was calculated as A_bound_/A_total_*100. The results were presented as average ± SD of 3 independent experiments.

**Results:** The results are reported in the main article and Table S5

**Table S5** Binding to serum proteins of ^67^Ga- and ^44^Sc-labeled lisinopril derivatives

| **Radioconjugate** | **Fraction bound to mouse blood plasma proteins** | **Fraction bound to human blood plasma proteins** |
| --- | --- | --- |
| [^67^Ga]Ga-DOTA-LIS-01 | 46 ± 3% | 45 ± 1% |
| [^67^Ga]Ga-DOTA-LIS-02 | 46 ± 1% | 50 ± 1% |
| [^67^Ga]Ga-NODAGA-LIS-02 | 49 ± 4% | 54 ± 3% |
| [^44^Sc]Sc-DOTA-LIS-02 | 42 ± 4% | 49 ± 2% |

Results are shown as average ± SD of n = 3 independent experiments

**6. Determination of cell uptake of lisinopril radioconjugates**

**Purpose:** In order to investigate the binding specificity of the lisinopril radioconjugates, cell binding experiments were performed in HEK-ACE and HEK-ACE2 cells.

**Methods:** Uptake and internalization of lisinopril radioconjugates were determined using HEK-ACE and HEK-ACE2 cells. The cells were seeded in poly-d-lysine-coated 12-well plates, allowing cell adhesion and growth overnight. After removal of the supernatant, the cells were incubated with fresh medium and the radioconjugates (25 µL, 1.9 pmol, 38 kBq) in the absence and presence of excess lisinopril (4 µM) for 1 h and 3 h. Afterwards, the cells were rinsed with PBS or acidic stripping buffer (glycine buffer with NaCl 0.9%, pH 2.8) to determine the total uptake and internalized fraction, respectively. An aqueous NaOH solution (1 M, 1 mL) was used to lyse the cells followed by transfer to tubes for counting the activity using a γ-counter (Wallac Wizard 1480, PerkinElmer). The results were expressed as a percentage of total added activity and normalized to an average content of ~0.4 mg protein per well. Statistical analysis was performed by applying a paired t-test to characterize the effect of linker length, the chelator type and the radiometal, respectively, on the binding to ACE of lisinopril radioconjugates (GraphPad Prism software, version 8.3.1).

**Results:** The results are reported in the main article.

**7. Determination of the relative binding affinity to ACE**

**Purpose:** In order to determine the relative ACE-binding affinity of ^67^Ga-labeled radioconjugates, saturation binding curves were performed on HEK-ACE cells to obtain the respective K_D_ values.

**Methods**: In order to determine the ACE-binding affinities of [^67^Ga]Ga-DOTA-LIS-01, [^67^Ga]Ga-DOTA-LIS-02 and [^67^Ga]Ga-NODAGA-LIS-02, the respective radioconjugates were labeled with gallium-67 (5 MBq/nmol) as previously described. In this case, however, a slight excess of ^nat^GaCl_3_ was added after successful radiolabeling before heating a second time to 95 °C for 10 min. This allowed to obtain pure Ga-complexes based on a mixture of conjugates coordinated with the gallium-67 and stable (natural) gallium. A suspension of HEK-ACE cells (2.5×10^5^ cells/0.5 ml) was prepared and incubated with increasing concentrations (1‒2000 nM, four samples per concentration) of the radioconjugate at 37 °C on a shaker. Half of the cell samples were co-incubated with an excess of lisinopril (20 µM, Tokyo Chemical Industry) to block ACE, enabling the determination of non-specific binding of the Ga-(radio)conjugates. After 1 h incubation, the cell samples were centrifuged (1000 rcf) for 5 min. The supernatant removed and the cell pellet resuspended in ice-cold PBS (pH 7.4, 500 µL). After centrifugation (1000 rcf) for 5 min, the cells were lysed with NaOH (1 M, 600 µL) and counted for activity using a γ-counter (Wallac Wizard 1480, PerkinElmer). The values obtained from the samples co-incubated with an excess of lisinopril were deducted from the other samples (total binding) to obtain values for the specific binding. Non-linear regression analysis was performed using GraphPad Prism software (version 8.3.1) to obtain the K_D_ values from three independent experiments each obtained from cell samples in duplicates (4 samples per concentration of radioconjugate of which two were co-incubated with excess lisinopril). The receptor-binding affinities were reported as inverse K_D_ values set in relation to the value obtained for [^67^Ga]Ga-DOTA-LIS-02 which was set as 1.0. The relative binding affinities were reported as the average ± SD of n = 3 independent experiments.

**Results:** The results are reported in the main article and listed in Table S6.

**Table S6** Relative binding affinity of ^67^Ga/^nat^Ga-labeled lisinopril conjugates compared to the receptor-binding affinity of [^67^Ga]Ga-DOTA-LIS-02 (set as 1.0)

| **Metal (radio)conjugate** | **Relative ACE-binding affinity** |
| --- | --- |
| [^67^Ga]Ga-DOTA-LIS-01 | 0.42 ± 0.03 |
| [^67^Ga]Ga-DOTA-LIS-02 | 1.0 ± 0.3 |
| [^67^Ga]Ga-NODAGA-LIS-02 | 0.55 ± 0.09 |

Results are shown as average ± SD of n = 3 independent experiments

**8. Biodistribution studies of [^67^Ga]Ga-DOTA-LIS-02 in xenografted mice**

**Purpose:** Biodistribution studies of [^67^Ga]Ga-DOTA-LIS-02, [^67^Ga]Ga-NODAGA-LIS-02 and [^44^Sc]Sc-DOTA-LIS-02 were performed in HEK-ACE/ACE2 xenografted nude mice to determine the pharmacokinetic profile of these radioconjugates.

**Methods:** Detailed methods are reported in the main article.

**Results:** Biodistribution data are presented and discussed in the main article, and the individual values are listed in Table S7.

**Table S7** Biodistribution data of [^67^Ga]Ga-DOTA-LIS-02, [^67^Ga]Ga-NODAGA-LIS-02 and [^44^Sc]Sc-DOTA-LIS-02 in HEK-ACE xenografted CD1/nude mice

|  | **[^67^Ga]Ga-DOTA-LIS-02** | | **[^67^Ga]Ga-NODAGA-LIS-02** | | **[^44^Sc]Sc-DOTA-LIS-02** | |
| --- | --- | --- | --- | --- | --- | --- |
|  | **1 h p.i.** | **3 h p.i.** | **1 h p.i.** | **3 h p.i.** | **1 h p.i.** | **3 h p.i.** |
|  | [%IA/g] | [%IA/g] | [%IA/g] | [%IA/g] | [%IA/g] | [%IA/g] |
| Blood | 0.35 ± 0.13 | <0.05 | <0.05 | <0.05 | 0.24 ± 0.03 | <0.05 |
| Heart | 0.20 ± 0.06 | <0.05 | <0.05 | <0.05 | 0.18 ± 0.03 | <0.05 |
| Lung | 2.4 ± 0.7 | 0.21 ± 0.05 | 0.63 ± 0.06 | 0.11 ± 0.04 | 2.0 ± 0.6 | 0.13 ± 0.02 |
| Spleen | 0.51 ± 0.65 | 0.05 ± 0.01 | <0.05 | <0.05 | 0.14 ± 0.05 | 0.06 ± 0.01 |
| Kidneys | 6.3 ± 1.0 | 1.1 ± 0.2 | 1.7 ± 0.6 | 0.49 ± 0.13 | 6.1 ± 0.8 | 0.75 ± 0.07 |
| Stomach | 0.16 ± 0.05 | 0.16 ± 0.18 | 0.31 ± 0.18 | 0.15 ± 0.10 | 0.22 ± 0.15 | <0.05 |
| Intestines | 0.33 ± 0.12 | 0.22 ± 0.90 | 23 ± 6 | 2.3 ± 1.0 | 0.16 ± 0.02 | 0.05 ± 0.01 |
| Liver | 2.0 ± 1.5 | 0.76 ± 0.13 | 18 ± 1 | 9.6 ± 1.2 | 0.14 ± 0.01 | 0.09 ± 0.01 |
| Muscle | 0.10 ± 0.03 | <0.05 | 0.11 ± 0.12 | <0.05 | 0.16 ± 0.02 | 0.07 ± 0.04 |
| Bone | 0.14 ± 0.01 | <0.05 | <0.05 | <0.05 | 0.17 ± 0.02 | 0.07 ± 0.03 |
| HEK-ACE2 xgft | 0.24 ± 0.01 | 0.08 ± 0.03 | 0.08 ± 0.01 | <0.05 | n.d. | n.d. |
| HEK-ACE xgft | 14 ± 3 | 16 ± 1 | 3.6 ± 0.9 | 5.2 ± 0.5 | 11 ± 2 | 8.1 ± 0.4 |
| Salivary glands | 0.18 ± 0.04 | <0.05 | <0.05 | <0.05 | 0.19 ± 0.02 | 0.09 ± 0.05 |
| HEK-ACE-to-kidney | 2.1 ± 0.2 | 14 ± 1 | 2.2 ± 0.2 | 11 ± 2 | 1.8 ± 0.1 | 11 ± 1 |
| HEK-ACE-to-blood | 34 ± 1 | 447 ± 21 | 83 ± 1 | 635 ± 253 | 46 ± 10 | 565 ± 145 |
| HEK-ACE-to-liver | 12 ± 1 | 21 ± 3 | 0.21 ± 0.02 | 0.55 ± 0.05 | 77 ± 10 | 86 ± 7 |

Decay-corrected biodistribution data shown as % IA/g tissue, representing the average ± SD of n = 3 mice; n.d. = not determined

**9. SPECT/CT and PET/CT imaging studies**

**Purpose:** SPECT/CT or PET/CT imaging studies were performed with HEK-ACE/ACE2 xenograft-bearing mice to evaluate the whole body tissue distribution of the lisinopril radioconjugates.

**Methods:** SPECT/CT imaging was performed using a four-head, multiplexing, multipinhole small-animal SPECT camera (NanoSPECT/CT™, Mediso Medical Imaging Systems, Budapest, Hungary). Each head was outfitted with a tungsten-based aperture of nine 1.4 mm-diameter pinholes and a thickness of 10 mm. CT scans of 7.5 min duration were followed by SPECT scans of ~50 min performed at 1 h, 3 h and 24 h after injection of 10 MBq [^67^Ga]Ga-DOTA-LIS-02 or 10 MBq [^67^Ga]Ga-NODAGA-LIS-02 (0.5 nmol, 100 μL), diluted in saline containing 0.05% BSA. During the SPECT/CT scan, mice were anesthetized with a mixture of isoflurane (1.5–2.0%) and oxygen. The images were acquired using Nucline software (version 1.02, Mediso Ltd., Budapest, Hungary). The real-time CT reconstruction used a cone-beam filtered back projection. The reconstruction of SPECT data was performed with HiSPECT software (version 1.4.3049, Scivis GmbH, Göttingen, Germany) using γ-energies of 93.20 keV (± 10%), 184.60 keV (± 10%) and 300.00 keV (± 10%) for gallium-67. All images were prepared using the VivoQuant post-processing software (version 3.5, inviCRO Imaging Services and Software, Boston, USA). A Gauss post-reconstruction filter (full width at half maximum, 1 mm) was applied, and the scale of activity set as indicated on the SPECT/CT images.

PET/CT imaging was performed using a small-animal PET/CT scanner (G8 PET/CT; Xodus Imaging CA, USA [1]). Static whole body PET scans of 10 min duration were performed at 1 h and 3 h after intravenous injection of 3 MBq [^44^Sc]Sc-DOTA-LIS-02 (3 MBq, 1 nmol, 100 µL) diluted in saline containing 0.05% BSA. The PET scan was followed by a CT scan of 1.5 min duration. During PET/CT acquisitions, the mice were anesthetized using a mixture of isoflurane (1.5–2.0%) and oxygen. The acquisition of the data and their reconstruction was performed using the G8 PET/CT scanner software (version 2.0.0.10). The images were prepared using VivoQuant post-processing software (version 3.5, inviCRO Imaging Services and Software, USA). A Gauss post-reconstruction filter (full width at half maximum, 1 mm) was applied, and the scale of activity set as indicated on the PET/CT images.

**Results:** The results are presented and discussed in the main article.

**10. In vitro autoradiography study using [^67^Ga]Ga-DOTA-LIS-02**

**Purpose:** In vitro autoradiography studies were performed to assess the species cross-reactivity of the radioconjugates and confirm their ability to detect ACE at physiological expression levels.

**Method:** Autoradiography studies were performed with [^67^Ga]Ga-DOTA-LIS-02 (80 MBq/nmol) on 20-µm thick frozen tissue sections of HEK-ACE and HEK-ACE2 xenografts as well as on sections of the kidneys and lung embedded in Tissue-Tek O.C.T. and frozen at −80 °C. Sections were prepared using a cryostat (Epredia Cryostar NX70 Cryostat, Microm International GmbH, Dreieich, Germany) on slides (Superfrost^TM^, Plus Adhesion Microscope Slides, epredia). The sections were incubated in a Tris-HCl buffer (170 nM, pH 7.6, with 5 mM MgCl_2_) with 0.25% BSA for 10 min before being exposed to [^67^Ga]Ga-DOTA-LIS-02 (225 kBq/150 µL) in Tris-HCl buffer with 1% BSA for 60 min at RT with or without excess of lisinopril (10 µM). After incubation, the tissue sections were rinsed twice for 5 min with Tris-HCl buffer containing BSA followed by rinsing the sections with Tris-HCl buffer without BSA and shortly with pure Milli-Q water. After drying the sections at RT, images were obtained using a storage phosphor imager (Cyclone Plus, PerkinElmer) and quantified using OptiQuant software (version 5.0 Bright Instrument Co Ltd., PerkinElmer). The tissue sections were exposed together with 1‒3 µL of dilutions of the radioconjugate solution of known activities (15‒1500 Bq). The amount of activity bound to ACE on the tissue sections was quantified by converting the signal intensity measured as digital light unit (DLU)/mm^2^ to activity per area in Bq/mm^2^. The specific ACE binding of the radioconjugates on the xenografts and organ tissue section was obtained by subtracting the signal (Bq/mm^2^ value) from tissue incubated with an excess of lisinopril from the respective signals (Bq/mm^2^ values) of their adjacent sections. The presented data were obtained from two independent experiments performed in duplicates of normal organs and xenografts from n = 2‒3 mice.

**Results:** The results are reported in the main manuscript and in Fig. S5a.

**11. Immunohistochemical staining of ACE on xenograft and mouse tissue sections**

**Purpose**: Immunohistochemical staining was performed on paraffin-embedded tissue sections of HEK-ACE and HEK-ACE2 xenografts as well as of the kidneys and lungs of CD1/nude mice. The aim was to investigate the expression of human and mouse ACE, respectively, in these tissues.

**Methods:** Immunohistochemistry was performed on 2-µm thick sections of formalin-fixed paraffin-embedded tissue at the University Hospital Zurich, Switzerland. Immunohistochemical detection of ACE was performed with a recombinant monoclonal rabbit anti-ACE antibody (EPR2757; Abcam Cambridge, UK; applied dilution 1:200). The immunohistochemical detection of human ACE was performed using a Ventana BenchMark automated staining system (Roche Diagnostics, Rotkreuz, Switzerland) using the optiView DAB IHC detection kit (Roche Diagnostics, Rotkreuz, Switzerland). Mouse ACE was detected using the BOND Polymer Refine Detection kit (Biosystems Switzerland AG, Muttenz, Switzerland) and the immunohistochemical staining was performed using a Leica BOND RX system (Biosystems Switzerland AG, Muttenz, Switzerland) .

**Results:** The results are reported in the main manuscript and representative images are shown in Fig. S5b. The immunohistochemical staining of HEK-ACE xenograft tissue sections revealed pronounced membranous expression of ACE, while no signal was observed on sections of ACE-negative HEK-ACE2 xenografts, indicating the specificity of the applied antibody for ACE. In renal tissue, ACE expression was primarily localized in the tubular epithelium with no detectable expression in the glomerular epithelium. In contrast, the lung tissue exhibited pronounced ACE expression on alveolar endothelial cells while epithelial cells of the alveoli and bronchus showed no detectable expression. The HEK-ACE xenograft expressed considerably higher levels of ACE than was the case in physiological mouse tissues.

**
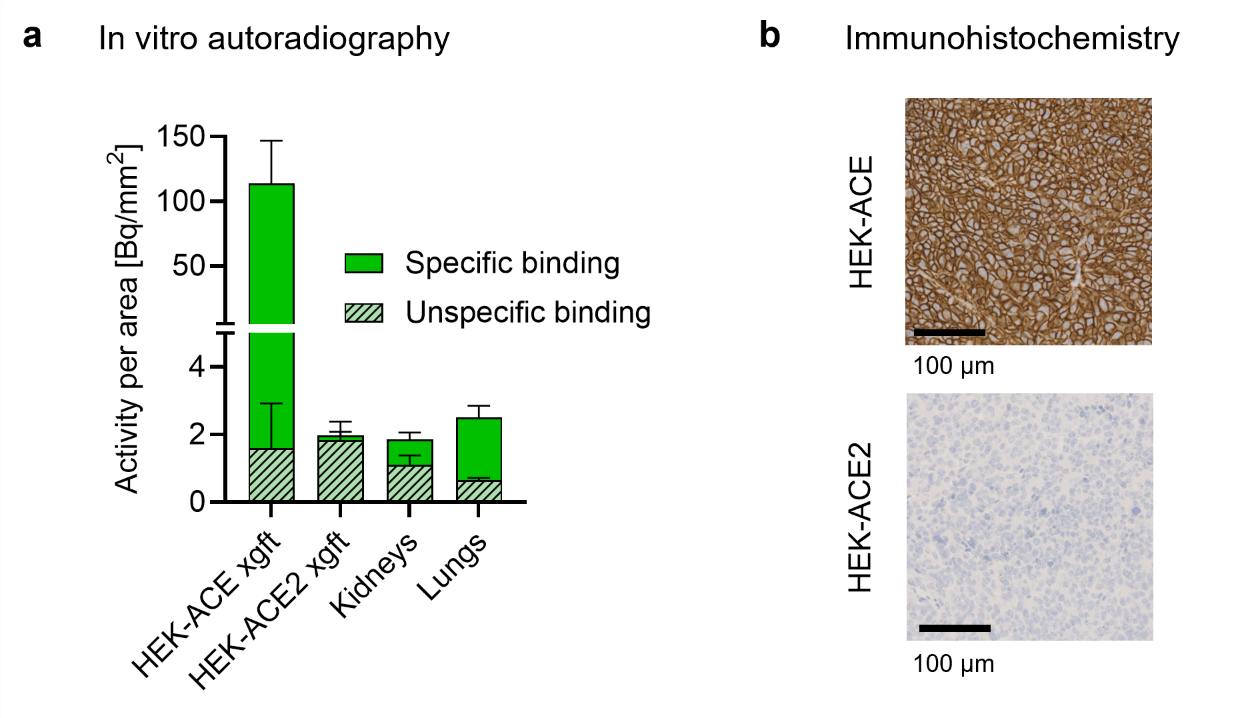
**

**Fig. S5** **a** Quantification of autoradiograms performed with [^67^Ga]Ga-DOTA-LIS-02 on frozen sections of mouse tissue including HEK-ACE and HEK-ACE2 xenografts, kidneys and lungs. **b** Representative imunohistochemical staining of ACE on HEK-ACE and HEK-ACE2 xenograft tissue.

**12. Computational studies**

**Purpose**: Molecular docking and molecular dynamics simulations were performed on Ga-DOTA-LIS-01 and Ga-DOTA-LIS-02 complexed with the ACE protein to estimate their binding modes and explain the differences in the relative affinities.

**Methods:** All the computational simulations were carried out on a 32 Core AMD Ryzen 93, 905×, 3.5 GHz Linux Workstation (O.S. Ubuntu 20.04) equipped with GPU (Nvidia Quadro RTX 4000, 8 GB). The structures of Ga-DOTA-LIS-01 and Ga-DOTA-LIS-02 were prepared with Avogadro software (version 1.2.0) [2]. The 3D-conformation of lisinopril moiety was derived from the Protein Data Bank (PDB; PDB-ID 1O86). The 3D-conformation of the Ga-DOTA moiety was derived from the Cambridge Crystallographic Data Center (CCDC-ID: 101668). The 3D-structure of the C-domain of ACE was derived from literature (PDB-ID: 4CA5; residues 40-617). Missing side chains were modelled and hydrogen atoms were added using the UCSF Chimera software (version 1.17.3) [3]. Docking simulations were conducted with the AutoDock Vina software (version 1.2.3) [4] using a docking box of 28x44x28 Å dimensions and setting exhaustiveness to 32. To obtain faster and reliable results, the bonds in the lisinopril and in the DOTA fragments were set unrottable when preparing the appropriate “.pdbqt” input files for AutoDock Vina based docking. Zinc and gallium atoms were excluded from the computation of binding mode. The lowest energy conformations were used for both conjugates as input for the molecular dynamics simulations. Molecular dynamics were conducted using Gromacs (version 2021.1) [5, 6], under the Charmm36 force field. Zinc and gallium ions were included in the simulations using standard parameters of the Charmm36 force field. The parameters for Ga-DOTA-LIS-01 and Ga-DOTA-LIS-02 were obtained through the CGenFF website [7]. To ensure that periodic images did not interact each other during the simulations, either ACE/Ga-DOTA-LIS-01 or ACE/Ga-DOTA-LIS-02 were included in cubic simulation boxes of 10.5x10.5x10.5 nm length. Complexes were hydrated with TIP3P water and neutralized using 0.15 M NaCl. In this way, the systems comprised the following components: 1 protein, 1 ligand, 34670 water molecules, 118 sodium and 104 chloride ions. Systems were equilibrated for 100 ps in the NVT ensemble and for 100 ps in the NPT ensemble. Long range electrostatic interactions were modelled using the Particle Mesh Ewald algorithm with a cut-off of 1.2 nm. LINCS, Nosé-Hoover and Parrinello-Rahman algorithms were used in the simulations for restraints, and as thermostat and barostat, respectively. Molecular dynamics were conducted for 100 nanoseconds with 2fs time steps. Root mean square deviation (RMSD) and root-mean-square-fluctuation (RMSF) values, short-range Coulomb and Lennard-Jones interaction energies and number of intermolecular hydrogen bonds were calculated using the functions implemented in Gromacs.

**Results:** The results are reported in the main article and in Figs S6 a-f. The high-resolution video depicting the entire molecular dynamic simulation was uploaded to th Zenodo platform (DOI: 10.5281/zenodo.15311169).

**
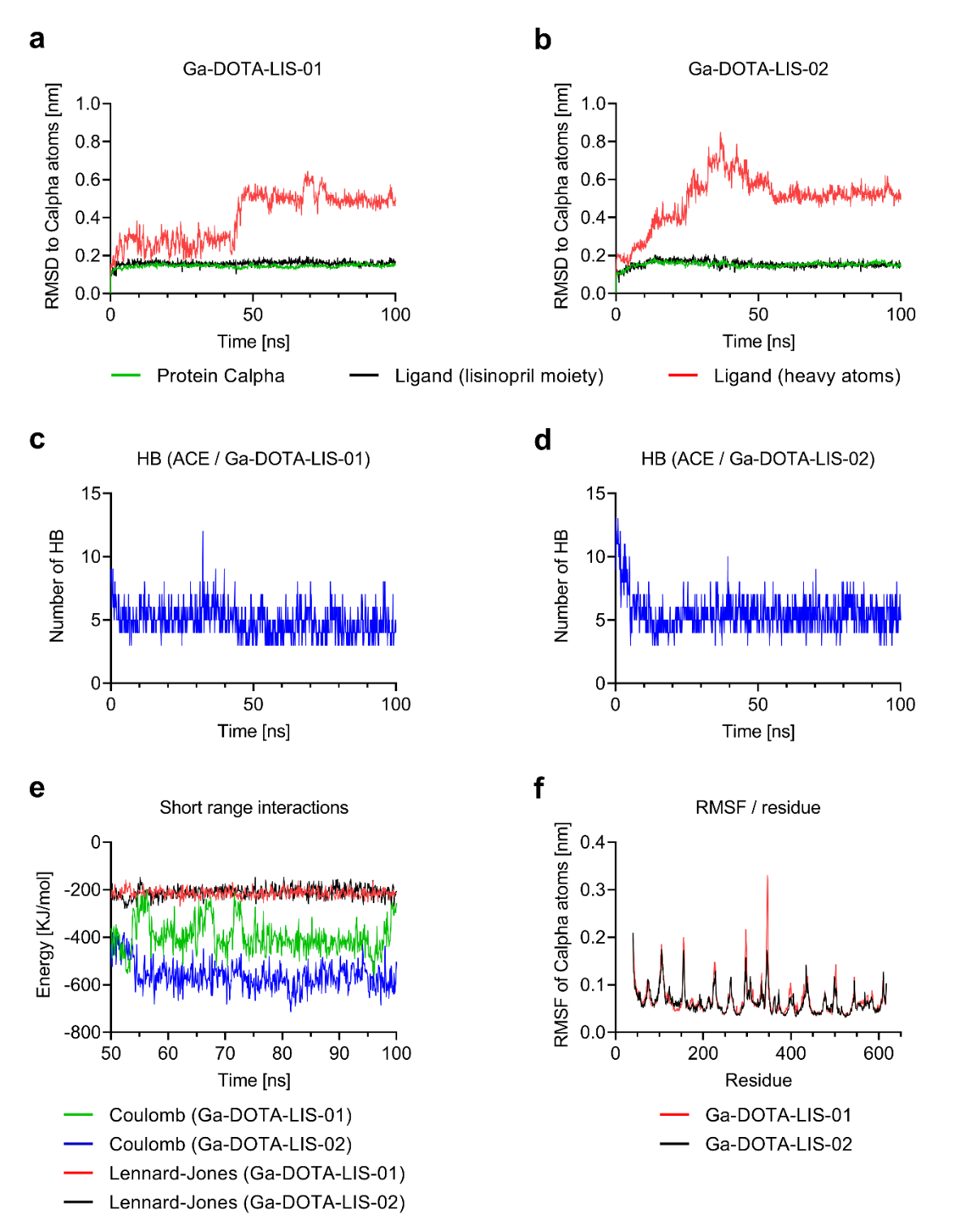
**

**Fig. S6** **a-f** Results from 100 ns of unbiased molecular dynamics simulations. (**a**/**b**) Root-mean-square-deviation (RMSD) of atomic position values (nm) for the protein Calpha and heavy atoms and lisinopril moiety of the ligands. (**c**/**d**) Number of hydrogen bonds (HB) formed between ACE and the ligands (HB with water were not included in the computation). (**e**) Comparison of short-range Coulomb and Lennard-Jones interaction energies between ACE and ligands (interaction energies with water were not included in the computation) in the last 50 ns of simulation (50–100 ns). (**f**) Comparison of Calpha root-mean-squared-fluctuation (RMSF) as function of residue number for ACE bound to Ga-DOTA-LIS-01 and Ga-DOTA-LIS-02 in the last 50 ns of simulation (50–100 ns)

**References**

1. Gu Z, Taschereau R, Vu NT, Prout DL, Silverman RW, Lee JT, et al. Performance evaluation of G8, a high-sensitivity benchtop preclinical PET/CT tomograph. J Nucl Med 2019;60:142-9. doi:10.2967/jnumed.118.208827.

2. Hanwell MD, Curtis DE, Lonie DC, Vandermeersch T, Zurek E, Hutchison GR. Avogadro: an advanced semantic chemical editor, visualization, and analysis platform. J Cheminform. 2012;4:17. doi:10.1186/1758-2946-4-17.

3. Pettersen EF, Goddard TD, Huang CC, Couch GS, Greenblatt DM, Meng EC, et al. UCSF Chimera--a visualization system for exploratory research and analysis. J Comput Chem. 2004;25:1605-12. doi:10.1002/jcc.20084.

4. Eberhardt J, Santos-Martins D, Tillack AF, Forli S. AutoDock Vina 1.2.0: new docking methods, expanded force field, and python bindings. J Chem Inf Model. 2021;61:3891-8. doi:10.1021/acs.jcim.1c00203.

5. Pronk S, Pall S, Schulz R, Larsson P, Bjelkmar P, Apostolov R, et al. GROMACS 4.5: a high-throughput and highly parallel open source molecular simulation toolkit. Bioinformatics. 2013;29:845-54. doi:10.1093/bioinformatics/btt055.

6. van der Spoel D, Lindahl E, Hess B, Groenhof G, Mark AE, Berendsen HJ. GROMACS: fast, flexible, and free. J Comput Chem. 2005;26:1701-18. doi:10.1002/jcc.20291.

7. Vanommeslaeghe K, Hatcher E, Acharya C, Kundu S, Zhong S, Shim J, et al. CHARMM general force field: A force field for drug-like molecules compatible with the CHARMM all-atom additive biological force fields. J Comput Chem. 2010;31:671-90. doi:10.1002/jcc.21367.
